# Analytical Solutions for a Charged Particle with White, Thermal, and Active Noises in the Presence of a Uniform Magnetic Field

**DOI:** 10.3390/e28070766

**Published:** 2026-07-04

**Authors:** Yun Jeong Kang, Sung Kyu Seo, Kyungsik Kim

**Affiliations:** 1 School of Liberal Studies, Wonkwang University, Iksan 54538, Republic of Korea; yjkang66@wku.ac.kr; 2 Haena Ltd., Seogwipo 63568, Republic of Korea; ggaru79@gmail.com; 3 Department of Physics, Pukyong National University, Busan 48513, Republic of Korea; 4 DigiQuay Ltd., Anyang 14084, Republic of Korea

**Keywords:** Vlasov equation, trap force, random force, correlated Gaussian force, super-diffusion, normal diffusion

## Abstract

In this paper, we apply the double Fourier transform method to the two-dimensional Vlasov equations for a charged particle subjected to white noise, exponentially correlated Gaussian forces, trap forces and thermal and active noises in a magnetic field. By deriving the corresponding Fokker–Planck equation, analytical solutions for the joint probability density are obtained in different time domains. The mean squared displacement and velocity of a charged particle driven by white noise exhibits a super-diffusive behavior, scaling as $\sim t^{2}$ in the short-time regime, while it grows linearly with time (~t) in the long-time regime, in agreement with numerical simulations of the mean squared displacement. When thermal noise is included together with harmonic trap and viscous forces, the characteristic time scale increases as $\sim t^{2h+1}$ in the corresponding time domains, whereas the mean squared velocity scales as $\sim t^{2h+3}$. The moments of the joint probability density under thermal noise scale as ${\sim t}^{2h+5}$. Furthermore, when the persistent Hurst exponent h→1/2, the entropy of the joint probability density associated with thermal noise coincides with that obtained for active noise in both the short-time (t≪τ) and long-time (t≫τ) limits.

## 1. Introduction

The Vlasov–Fokker–Planck equation is a fundamental partial differential equation obtained by combining the Vlasov equation [1], which describes collisionless particle dynamics under long-range electromagnetic forces, with the Fokker–Planck equation, which models stochastic diffusion processes in velocity space. This equation governs the evolution of particle distribution functions in phase space for systems in which collective self-consistent fields coexist with weak collisions or dissipative effects. The Vlasov equation has been widely applied in statistical mechanics, plasma physics, particle beam dynamics, and even in certain biological systems.

Prior to the last four decades, the Chirikov–Taylor model [2,3] was extensively studied, and analytical solutions were developed for a wide range of plasma physics problems [4,5,6]. Probabilistic methods and powerful analytical techniques for solving the Vlasov equation were directly applied to this model. As a result, analytical expressions for the probability density function, its second velocity moment, and the associated spatial diffusion coefficient were obtained. In the framework of the Vlasov equation, a collisionless plasma is described by deterministic particle trajectories in a magnetic field. Each particle follows a spiral trajectory around the magnetic field lines determined by its initial conditions. Consequently, the mean squared displacement either oscillates around a constant value or converges to a finite limit set by the initial distribution, rather than diverging indefinitely. This behavior reflects the fact that particles remain confined by the magnetic field and do not undergo unbounded diffusion.

Jimenez-Aquino and Romero-Bastida [7] investigated diffusion in the Langevin discharge equation, incorporating the effects of magnetic fields and colored noise, and analyzed long-time diffusion behavior as well as the influence of magnetic fields on the mean squared displacement. Tothova and Lisy [8] studied the diffusion of charged particles using an exponentially correlated random force model, identifying distinct time-domain behaviors through linear and exponentially decaying contributions to the mean squared displacement. In a subsequent work, Tothova and Lisy [9] showed that subdiffusive behavior (the fractal dimension α=1/2) may emerge in the long-time limit within a generalized Langevin equation framework. More recently, we obtained analytical probability densities for active particles coupled to two heat reservoirs [10], passive particles subjected to harmonic and viscous forces [11], and passive particles in random environments under magnetic fields with correlated Gaussian forces [12]. In addition, we analyzed the dynamical behavior of an active Brownian particle confined by an optical trap and driven by correlated Gaussian noise [13].

The modified Vlasov model that includes magnetic fields is of particular interest because it can be extended to chaotic and turbulent stochastic dynamics. This extension becomes possible through the incorporation of correlated Gaussian forces, which allows new analytical solutions to be obtained using the Fourier transform method. The purpose of this paper is to present analytical solutions of the modified Vlasov equation. Specifically, we consider a charged particle in a magnetic field subjected to exponentially correlated Gaussian forces. The paper is organized as follows. In Section 2, we introduce the modified Vlasov equation for a charged particle in a magnetic field and derive the probability density functions for the displacement and velocity in the limits t<<τ, t>>τ and for τ=0, where τ denotes the correlation time. In Section 3, we analyze the Vlasov equation with exponentially correlated Gaussian forces in a magnetic field. Section 4 presents analytical solutions for a charged colloidal particle subjected to trap forces, thermal equilibrium noise $\zeta_{th}(t)$, and active noise $\zeta_{ac}(t)$ with exponentially decaying correlations. In Section 5, we compute statistical quantities such as the non-Gaussian parameter, correlation coefficients, entropy, and combined entropy. Finally, our conclusions are summarized in Section 6.

## 2. Modified Vlasov Equation

The general Vlasov equation [6], including nonrelativistic electric and magnetic fields, is given by

(1) $$\frac{\partial f}{\partial t}+v\cdot \nabla_{r}f+\frac{q}{m}\left(E+v\times B\right)\cdot \nabla_{v}f={D\nabla_{r}}^{2}f.$$

In the case where only the magnetic field $B=B_{z}\hat{z}$ exists in two dimensions, the probability density function in the Vlasov equation is written as

(2) $$\frac{\partial }{\partial t}f+v_{x}\frac{\partial }{\partial x}f+v_{y}\frac{\partial }{\partial y}f+\frac{q}{m}v_{y}B_{z}\frac{\partial }{\partial v_{x}}f-\frac{q}{m}v_{x}B_{z}\frac{\partial }{\partial v_{y}}f=D_{x}\frac{\partial^{2}}{{\partial x}^{2}}f+D_{y}\frac{\partial^{2}}{{\partial y}^{2}}f.$$

Here, the velocity is $v=v_{x}\hat{x}+v_{y}\hat{y}$, and $D_{x}$ and $D_{y}$ denote the spatial diffusion coefficients.

The equations of motion for a charged particle in the presence of $B_{z}\hat{z}$ are given by

(3) $$\frac{\partial }{\partial t}x=v_{x}{+\eta }_{x}\left(t\right),\;\;m\frac{\partial }{\partial t}v_{x}=-qv_{y}B_{z},$$

and

(4) $$\frac{\partial }{\partial t}y=v_{y}{+\eta }_{y}\left(t\right),\;m\frac{\partial }{\partial t}v_{y}=+qv_{x}B_{z}.$$

Here, the random forces $\eta_{x}(t)$ and $\eta_{y}(t)$ depend on the time difference and satisfy $<\eta_{i}\left(t\right)\eta_{i}\left(t^{\prime }\right)>=\frac{D_{i}}{\tau }\delta (\frac{\left|t-t\prime \right|}{\tau })$ for i=x,y. We show in Appendix A that the time derivative of the joint probability density derived from Equations (3) and (4) is consistent with Equation (2).

The joint probability densities $f(x,v_{x},t)$ and $f(y,v_{y},t)$ for the displacement x,y and the velocity $v_{x},v_{y}$ are defined by $f(x,v_{x},t)=<\delta (x-x(t))\delta (v_{x}-v_{x}(t))>$ and $f(y,v_{y},t)=<\delta (y-y(t))\delta (v_{y}-v_{y}(t))>$. By taking time derivatives of these joint probability densities, we have the differential equations

(5) $$\frac{\partial }{\partial t}f(x,v_{x},t)=-\frac{\partial }{\partial x}<\frac{\partial x}{\partial t}\delta (x-x(t))\delta (v_{x}-v_{x}(t))>-\frac{\partial }{\partial v_{x}}<\frac{\partial v_{x}}{\partial t}\delta (x-x(t))\delta (v_{x}-v_{x}(t))>,$$

(6) $$\frac{\partial }{\partial t}f\left(y,v_{y},t\right)=-\frac{\partial }{\partial y}<\frac{\partial y}{\partial t}\delta \left(y-y\left(t\right)\right)\delta \left(v_{y}-v_{y}\left(t\right)\right)>-\frac{\partial }{\partial v_{y}}<\frac{\partial v_{y}}{\partial t}\delta \left(y-y\left(t\right)\right)\delta \left(v_{y}-v_{y}\left(t\right)\right).$$

We then introduce the following equations for the evolution of the joint probability densities:

(7) $$\frac{\partial }{\partial t}f\left(x,v_{x},t\right)=[-v_{x}\frac{\partial }{\partial x}+v_{x}{B_{z}}^{2}\frac{\partial }{\partial v_{x}}]f\left(x,v_{x},t\right)+D_{x}\frac{\partial^{2}}{\partial x^{2}}f\left(x,v_{x},t\right)$$

(8) $$\frac{\partial }{\partial t}f\left(y,v_{y},t\right)=[-v_{y}\frac{\partial }{\partial y}+v_{y}{B_{z}}^{2}\frac{\partial }{\partial v_{y}}]f\left(y,v_{y},t\right)+D_{y}\frac{\partial^{2}}{\partial y^{2}}f\left(y,v_{y},t\right).$$

Substituting Equations (3) and (4) into Equations (5) and (6), we use Equation (12) of Ref. [13] for the two cases of random forces $\eta_{x}(t)$ and $\eta_{y}(t)$. Then, Equations (7) and (8) are derived. Here, the dimensionless parameters m=1 and q=1 are used. The derivation of Equations (7) and (8) is presented in Appendix B.

(9) $$f(\zeta_{1},\nu_{1},t)=\int_{-\infty }^{+\infty }dx\int_{-\infty }^{+\infty }dv_{x}exp(-i\zeta_{1}x-i\nu_{1}v_{x})f(x,v_{x},t),$$

(10) $$f(\zeta_{2},\nu_{2},t)=\int_{-\infty }^{+\infty }dy\int_{-\infty }^{+\infty }dv_{y}exp(-i\zeta_{2}y-i\nu_{2}v_{y})f(y,v_{y},t).$$

By applying the double Fourier transform to Equations (9) and (10), we obtain the variable-separating equations

(11) $$\frac{\partial }{\partial t}f\left(\zeta_{1},\nu_{1},t\right)=\left[\zeta_{1}-{B_{z}}^{2}\nu_{1}\right]\frac{\partial }{\partial \nu_{1}}f\left(\zeta_{1},\nu_{1},t\right)-D_{x}\zeta_{1}^{2}f\left(\zeta_{1},\nu_{1},t\right)+Af(\zeta_{1},\nu_{1},t),$$

(12) $$\frac{\partial }{\partial t}f\left(\zeta_{2},\nu_{2},t\right)=\left[\zeta_{2}-{B_{z}}^{2}\nu_{2}\right]\frac{\partial }{\partial \nu_{2}}f\left(\zeta_{2},\nu_{2},t\right)-D_{y}\zeta_{2}^{2}f\left(\zeta_{2},\nu_{2},t\right)-Af\left(\zeta_{2},\nu_{2},t\right),$$

where A is the separation constant, and the initial conditions are $x_{0}={\;y}_{0}=0$. and $v_{x0}=v_{y0}=0$.

### 2.1. Probability Densities in Steady State

In this subsection, we obtain the Fourier transforms of the probability density function given by Equations (13) and (14) for the modified Vlasov equation, Equation (9), describing a charged particle.

#### 2.1.1. f(x,t) and $f(v_{x},t)$ in the Short- and Long-Time Domains 

By taking $\frac{\partial }{\partial t}f(\zeta_{1},\nu_{1},t)=0$ for $\nu_{1}$ in the steady state, $f(\zeta_{1},\nu_{1},t)$ reduces to the stationary distribution $f^{st}(\zeta_{1},\nu_{1},t)$. The Fourier transformed equation for the steady probability density from Equation (11) is

(13) $$\left[\zeta_{1}-{B_{z}}^{2}\nu_{1}\right]\frac{\partial }{\partial \nu_{1}}f^{st}\left(\zeta_{1},\nu_{1},t\right)-D_{x}\zeta_{1}^{2}f^{st}\left(\zeta_{1},\nu_{1},t\right)+Af^{st}(\zeta_{1},\nu_{1},t)=0.$$

The first term of the left-hand side in Equation (15) is approximated as ${\left[\zeta_{1}-{B_{z}}^{2}\nu_{1}\right]}^{-1}={\zeta_{1}}^{-1}[1+\frac{{B_{z}}^{2}\nu_{1}}{\zeta_{1}}]$. The stationary solution with respect to $\nu_{1}$ is then given by

(14) $$f^{st}\left(\zeta_{1},\nu_{1},t\right)=exp[\frac{1}{\zeta_{1}}[D_{x}\zeta_{1}^{2}\nu_{1}-A\nu_{1}]+\frac{{B_{z}}^{2}}{{\zeta_{1}}^{2}}[D_{x}\zeta_{1}^{2}\frac{\nu_{1}^{2}}{2}-A\nu_{1}]].$$

Introducing $f\left(\zeta_{1},\nu_{1},t\right)=\Theta [t+\nu_{1}/[\zeta_{1}-B_{z}\nu_{2}]]f^{st}(\zeta_{1},\nu_{1},t)$ [13], we obtain

(15) $$f\left(\zeta_{1},\nu_{1},t\right)=exp[-D_{x}t\zeta_{1}^{2}-D_{x}{B_{z}}^{2}t^{2}\zeta_{1}^{2}/2-D_{x}{B_{z}}^{6}{t^{2}\nu }_{1}^{2}/2].$$

Using the inverse Fourier transform, the probability densities in the short-time domain when $1\ll {B_{z}}^{2}t$ are given by

(16) $$f(x,t)=[2\pi D_{x}{B_{z}}^{2}t^{2}{]}^{-1/2}exp[-\frac{x^{2}}{2D_{x}{B_{z}}^{2}t^{2}}],f\left(v_{x},t\right)=[2\pi D_{x}{B_{z}}^{6}t^{2}{]}^{-1/2}exp[-\frac{v_{x}^{2}}{2D_{x}{B_{z}}^{6}t^{2}}].$$

In the long-time domain when ${B_{z}}^{2}t\ll 1$, the probability densities are obtained as

(17) $$f(x,t)=[4\pi D_{x}t{]}^{-1/2}exp[-\frac{x^{2}}{4D_{x}t}],f\left(v_{x},t\right)=[2\pi D_{x}{B_{z}}^{6}t^{2}{]}^{-1/2}exp[-\frac{v_{x}^{2}}{2D_{x}{B_{z}}^{6}t^{2}}].$$

The mean squared quantities corresponding to Equations (16) and (17) are given by

(18) $$<x^{2}(t)>=D_{x}{B_{z}}^{2}t^{2},\;<v_{x}^{2}\left(t\right)>=D_{x}{B_{z}}^{6}t^{2},$$

in the short-time domain, and

(19) $$<x^{2}(t)>=2D_{x}t,\;<v_{x}^{2}\left(t\right)>=D_{x}{B_{z}}^{6}t^{2},$$

in the long-time domain. Therefore, Equations (16) and (17) correspond to two asymptotic regimes determined by the dominant contribution in Fourier space rather than to the limits t→0 and t→∞.

#### 2.1.2. f(y,t) and $f(v_{y},t)$ in the Short- and Long-Time Domains

Taking $\frac{\partial }{\partial t}f(\zeta_{2},\nu_{2},t)=0$ for $\nu_{2}$ in the steady state, $f(\zeta_{2},\nu_{2},t)$ becomes $f^{st}(\zeta_{2},\nu_{2},t)$. The Fourier transformed equation for the steady probability density function from Equation (8) is obtained as

(20) $$\left[\zeta_{2}-{B_{z}}^{2}\nu_{2}\right]\frac{\partial }{\partial \nu_{2}}f^{st}\left(\zeta_{2},\nu_{2},t\right)-D_{x}\zeta_{2}^{2}f^{st}\left(\zeta_{2},\nu_{2},t\right)-Af^{st}(\zeta_{2},\nu_{2},t)=0.$$

For Equation (22), using a similar procedure to Equations (14) and (15), we have

(21) $$f\left(\zeta_{2},\nu_{2},t\right)=exp[-D_{y}t\zeta_{2}^{2}-D_{y}{B_{z}}^{2}t^{2}\zeta_{2}^{2}/2-D_{y}{B_{z}}^{6}{t^{2}\nu }_{2}^{2}/2].$$

Using the inverse Fourier transform, the probability density in the short-time domain when $1\ll {B_{z}}^{2}t$ is calculated as

(22) $$f(y,t)=[2\pi D_{y}{B_{z}}^{2}t^{2}{]}^{-1/2}exp[-\frac{y^{2}}{2D_{y}{B_{z}}^{2}t^{2}}],f\left(v_{y},t\right)=[2\pi D_{y}{B_{z}}^{6}t^{2}{]}^{-1/2}exp[-\frac{v_{y}^{2}}{2D_{y}{B_{z}}^{6}t^{2}}].$$

In the long-time domain when $1\gg {B_{z}}^{2}t$, the probability density also is calculated as

(23) $$f(y,t)=[4\pi D_{y}t{]}^{-1/2}exp[-\frac{y^{2}}{4D_{y}t}],f\left(v_{y},t\right)=[2\pi D_{y}{B_{z}}^{6}t^{2}{]}^{-1/2}exp[-\frac{v_{y}^{2}}{2D_{y}{B_{z}}^{6}t^{2}}].$$

Thus, the mean squared quantities for f(y,t) and $f(v_{y},t)$ from Equations (14) and (15) are, respectively, given by

(24) $$<y^{2}(t)>=D_{y}{B_{z}}^{2}t^{2},\;<v_{y}^{2}\left(t\right)>=D_{y}{B_{z}}^{6}t^{2},$$

in the short-time domain, and

(25) $$<y^{2}(t)>=2D_{y}t,\;<v_{y}^{2}\left(t\right)>=D_{y}{B_{z}}^{6}t^{2},$$

in the long-time domain. Thus, if the diffusion coefficient $D_{x}$ is equal to $D_{y}$, then the mean square displacement and mean square velocity in the *x*- and *y*-axis directions are equal, as we have seen in Section 2.1.1 and Section 2.1.2. This means that the motion of the charged particles has a statistical isotropy in the *x*- and *y*-axis directions.

## 3. Modified Vlasov Equation with Exponential Correlated Gaussian Forces in the Magnetic Field

In this section, we solve the probability density function for the modified Vlasov equation, Equations (3) and (4), of a charged particle with two correlated Gaussian forces within the limits of t<<τ, t>>τ, and for τ=0, where τ is the correlation time.

The two modified equations of motion for a charged particle with $\vec{B}=B_{z}\hat{z}$ are given by

(26) $$\frac{\partial }{\partial t}x=v_{x}{+g}_{x}\left(t\right),m\frac{\partial }{\partial t}v_{x}=-qv_{y}B_{z}{-r_{1}v}_{x},$$

and

(27) $$\frac{\partial }{\partial t}y=v_{y}{+g}_{y}\left(t\right),\;m\frac{\partial }{\partial t}v_{y}=+qv_{x}B_{z}{-r_{2}v}_{y}.$$

Here, the exponential correlated Gaussian forces $g_{x}\left(t\right)$ and $g_{y}\left(t\right)$ depend on the time difference: $<g_{i}\left(t\right)g_{i}\left(t^{\prime }\right)>=\frac{D_{i}}{\tau }exp(-\frac{\left|t-t\prime \right|}{\tau })$ for i=x,y [13,14]. ${-r_{1}v}_{x}$ and ${-r_{2}v}_{y}$ are the viscous forces, respectively.

The joint probability densities $f(x,v_{x},t)$ and $f(y,v_{y},t)$ for the displacement x,y and the velocity $v_{x},v_{y}$ are defined as $f(x,v_{x},t)=<\delta (x-x(t))\delta (v_{x}-v_{x}(t))>$ and $f(y,v_{y},t)=<\delta (y-y(t))\delta (v_{y}-v_{y}(t))>$. By taking time derivatives of these joint probability densities, we obtain the following differential equations:

(28) $$\frac{\partial }{\partial t}f(x,v_{x},t)=-\frac{\partial }{\partial x}<\frac{\partial x}{\partial t}\delta (x-x(t))\delta (v_{x}-v_{x}(t))>-\frac{\partial }{\partial v_{x}}<\frac{\partial v_{x}}{\partial t}\delta (x-x(t))\delta (v_{x}-v_{x}(t))>,$$

(29) $$\frac{\partial }{\partial t}f\left(y,v_{y},t\right)=-\frac{\partial }{\partial y}<\frac{\partial y}{\partial t}\delta \left(y-y\left(t\right)\right)\delta \left(v_{y}-v_{y}\left(t\right)\right)>-\frac{\partial }{\partial v_{y}}<\frac{\partial v_{y}}{\partial t}\delta \left(y-y\left(t\right)\right)\delta \left(v_{y}-v_{y}\left(t\right)\right).$$

Substituting Equation (26) into Equation (28) and Equation (27) into Equation (29), and following the procedure of Ref. [13], we obtain

(30) $$\frac{\partial }{\partial t}f\left(x,v_{x},t\right)=\left[-v_{x}\frac{\partial }{\partial x}+[-B_{z}v_{y0}e^{-r_{2}t}+{{(B}_{z}}^{2}{+r_{1})v}_{x}]\frac{\partial }{\partial v_{x}}\right]f\left(x,v_{x},t\right)+D_{x}[{-b}_{1}\left(t\right)\frac{\partial^{2}}{\partial x^{2}}+a_{1}(t)\frac{\partial^{2}}{\partial \nu_{x}^{2}}]f(x,v_{x},t),$$

(31) $$\frac{\partial }{\partial t}f\left(y,v_{y},t\right)={[-v}_{y}\frac{\partial }{\partial y}+[B_{z}v_{x0}e^{-r_{1}t}+{{(B}_{z}}^{2}{+r_{2})v}_{y}]\frac{\partial }{\partial v_{y}}]f\left(y{,v}_{y},t\right)+D_{y}[{-b}_{2}\left(t\right)\frac{\partial^{2}}{\partial y^{2}}+a_{2}(t)\frac{\partial^{2}}{\partial \nu_{y}^{2}}]f\left(y,v_{y},t\right).$$

Here, we have set the dimensionless parameters m=1 and q=1. The time-dependent coefficients are given by $a_{1}(t)=a_{2}(t)=1-exp(-t/\tau )$, $b_{1}(t)=b_{2}(t)=(t+\tau )exp(-t/\tau )-\tau$. Applying the double Fourier transform defined in Equations (9) and (10), we obtain the Fourier-space equations

(32) $$\frac{\partial }{\partial t}f\left(\zeta_{1},\nu_{1},t\right)=\left[\zeta_{1}-{{(B}_{z}}^{2}+r_{1})\nu_{1}\right]\frac{\partial }{\partial \nu_{1}}f\left(\zeta_{1},\nu_{1},t\right)+D_{x}[b_{1}(t)\zeta_{1}\nu_{1}-a_{1}(t)\zeta_{1}^{2}]f(\zeta_{1},\nu_{1},t),$$

(33) $$\frac{\partial }{\partial t}f\left(\zeta_{2},\nu_{2},t\right)=\left[\zeta_{2}-{{(B}_{z}}^{2}{+r_{2})\nu }_{2}\right]\frac{\partial }{\partial \nu_{2}}f\left(\zeta_{2},\nu_{2},t\right)+D_{y}[b_{2}(t)\zeta_{2}\nu_{2}-a_{2}(t)\zeta_{2}^{2}]f(\zeta_{2},\nu_{2},t).$$

### 3.1. Probability Densities in Three-Time Domains

#### 3.1.1. f(x,t), $f(v_{x},t)$ and f(y,t), $f(v_{y},t)$ in the Short-Time Limit t≪τ

In the short-time domain t≪τ, the steady-state solution of Equation (32) for fixed $\zeta_{1}$ is obtained as

(34) $$f^{st}(\zeta_{1},\nu_{1},t)=exp[\frac{D_{x}}{\zeta_{1}}\left[b_{1}\left(t\right)\zeta_{1}\frac{\nu_{1}^{2}}{2}-a_{1}\left(t\right)\zeta_{1}^{2}\nu_{1}\right]+\frac{{{D_{x}(B}_{z}}^{2}+r_{1})}{{\zeta_{1}}^{2}}[b_{1}\left(t\right)\zeta_{1}\frac{\nu_{1}^{3}}{3}-a_{1}(t)\zeta_{1}^{2}\frac{\nu_{1}^{2}}{2}]],$$

which yields the explicit exponential form given in Equation (34). To proceed, we introduce the successive transformations

(35) $$f\left(\zeta_{1},\nu_{1},t\right)=g\left(\zeta_{1},\nu_{1},t\right)f^{st}\left(\zeta_{1},\nu_{1},t\right),$$

(36) $$g\left(\zeta_{1},\nu_{1},t\right)=h\left(\zeta_{1},\nu_{1},t\right)exp[\frac{D_{x}}{\zeta_{1}^{2}}[b_{1}^{\prime }\left(t\right)\zeta_{1}\frac{\nu_{1}^{3}}{6}-a_{1}^{\prime }(t)\zeta_{1}^{2}\frac{\nu_{1}^{2}}{2}]+\frac{{{D_{x}(B}_{z}}^{2}+r_{1})}{\zeta_{1}^{3}}[b_{1}^{\prime }\left(t\right)\zeta_{1}\frac{\nu_{1}^{4}}{12}-a_{1}^{\prime }(t)(t)\zeta_{1}^{2}\frac{\nu_{1}^{3}}{6}]],$$

(37) $$h(\zeta_{1},\nu_{1},t)=p(\zeta_{1},\nu_{1},t)exp[\frac{D_{x}}{\zeta_{1}^{3}}[b_{1}^{\prime \prime }\left(t\right)\zeta_{1}\frac{\nu_{1}^{4}}{12}-a_{1}^{\prime \prime }(t)\zeta_{1}^{2}\frac{\nu_{1}^{3}}{6}]+\frac{{{D_{x}(B}_{z}}^{2}+r_{1})}{\zeta_{1}^{4}}[b_{1}^{\prime \prime }\left(t\right)\zeta_{1}\frac{\nu_{1}^{5}}{60}-{a\prime }_{1}^{\prime }(t)(t)\zeta_{1}^{2}\frac{\nu_{1}^{4}}{12}]],$$

(38) $$p(\zeta_{1},\nu_{1},t)=q(\zeta_{1},\nu_{1},t)exp[\frac{D_{x}}{\zeta_{1}^{4}}[b_{1}^{\prime \prime \prime }\left(t\right)\zeta_{1}\frac{\nu_{1}^{5}}{60}]+\frac{{{D_{x}(B}_{z}}^{2}+r_{1})}{\zeta_{1}^{5}}[b_{1}^{\prime \prime \prime }\left(t\right)\zeta_{1}\frac{\nu_{1}^{6}}{360}]].$$

where higher-order terms proportional to $1/\tau^{3}$ are neglected. Under this approximation, $q\left(\zeta_{1},\nu_{1},t\right)$ satisfies

(39) $$\frac{\partial }{\partial t}q(\zeta_{1},\nu_{1},t)=[\zeta_{1}-(B_{z}^{2}+r_{1})\nu_{1}]\frac{\partial }{\partial \nu_{1}}q(\zeta_{1},\nu_{1},t),$$

Expanding up to second order in t/τ and collecting all contributions, we obtain

(40) $$f\left(\zeta_{1},\nu_{1},t\right)=exp[-\frac{D_{x}t^{3}}{2}{\zeta_{1}}^{2}-\frac{{{D_{x}B}_{z}}^{4}t^{3}}{2}{\nu_{1}}^{2}].$$

The probability densities for displacement and velocity are then given by

(41) $$f(x,t)=[2\pi D_{x}t^{3}{]}^{-\frac{1}{2}}exp[-\frac{x^{2}}{2D_{x}t^{3}}],\;f(v_{x},t)=[2\pi {{D_{x}B}_{z}}^{4}t^{3}{]}^{-1/2}exp[-\frac{v_{x}^{2}}{{{D_{x}B}_{z}}^{4}t^{3}}],$$

with mean squared values

(42) $$<x^{2}(t)>=D_{x}t^{3},<v_{x}^{2}(t)>={{D_{x}B}_{z}}^{4}t^{3}.$$

Applying the same procedure to Equation (36), we obtain

(43) $$f\left(\zeta_{2},\nu_{2},t\right)=exp[-\frac{D_{y}t^{3}}{2}{\zeta_{2}}^{2}-\frac{{{D_{y}B}_{z}}^{4}t^{3}}{2}{\nu_{2}}^{2}],$$

leading to

(44) $$f(y,t)=[2\pi D_{y}t^{3}{]}^{-\frac{1}{2}}exp[-\frac{y^{2}}{2D_{y}t^{3}}],f(v_{y},t)=[2\pi {{D_{y}B}_{z}}^{4}t^{3}{]}^{-\frac{1}{2}}exp[-\frac{v_{y}^{2}}{{{D_{y}B}_{z}}^{4}t^{3}}].$$

Consequently,

(45) $$<y^{2}(t)>=D_{y}t^{3},\;<v_{y}^{2}(t)>={{D_{y}B}_{z}}^{4}t^{3}.$$

#### 3.1.2. f(x,t)*,* $f(v_{x},t)$ and f(y,t)*,* $f(v_{y},t)$ in t≫τ

In this subsection, we analyze the probability densities $f(\zeta_{1},\nu_{1},t)$ and $f(\zeta_{2},\nu_{2},t)$ in the long-time domain t≫τ. In this limit, Equations (31) and (32) can be approximated as

(46) $$\frac{\partial }{\partial t}f\left(\zeta_{1},\nu_{1},t\right)≅D_{x}[b_{1}(t)\zeta_{1}\nu_{1}-a_{1}(t)\zeta_{1}^{2}]f(\zeta_{1},\nu_{1},t),$$

(47) $$\frac{\partial }{\partial t}f\left(\zeta_{2},\nu_{2},t\right)≅D_{y}[b_{2}(t)\zeta_{2}\nu_{2}-a_{2}(t)\zeta_{1}^{2}]f(\zeta_{2},\nu_{2},t),$$

From these equations, we formally obtain

(48) $$f_{\zeta_{1}}\left(\zeta_{1},\nu_{1},t\right)=exp[D_{x}\int [b_{1}(t)\zeta_{1}\nu_{1}-a_{1}(t)\zeta_{1}^{2}]dt],$$

(49) $$f_{\zeta_{2}}\left(\zeta_{2},\nu_{2},t\right)=exp[D_{y}\int [b_{2}(t)\zeta_{2}\nu_{2}-a_{2}(t)\zeta_{1}^{2}]dt].$$

Using the relations $f(\zeta_{1},\nu_{1},t)=g_{\zeta_{1}}(\zeta_{1},\nu_{1},t)f_{\zeta_{1}}(\zeta_{1},\nu_{1},t)$ and $f(\zeta_{2},\nu_{2},t)=g_{\zeta_{2}}(\zeta_{2},\nu_{2},t)f_{\zeta_{2}}(\zeta_{2},\nu_{2},t)$, the steady-state factors are obtained as

(50) $$g_{\zeta_{1}}^{st}\left(\zeta_{1},\nu_{1},t\right)=exp[-D_{x}\int [b_{1}(t)\zeta_{1}\nu_{1}-a_{1}(t)\zeta_{1}^{2}]dt],$$

(51) $$g_{\zeta_{2}}^{st}\left(\zeta_{2},\nu_{2},t\right)=exp[-D_{y}\int [b_{2}(t)\zeta_{2}\nu_{2}-a_{2}(t)\zeta_{1}^{2}]dt].$$

The steady-state solutions $f^{st}(\zeta_{1},\nu_{1},t)$ and $f^{st}(\zeta_{2},\nu_{2},t)$, given in Equation (33), are rewritten as

(52) $$f^{st}\left(\zeta_{1},\nu_{1},t\right)=exp[\frac{D_{x}}{\zeta_{1}}[b_{1}\left(t\right)\zeta_{1}\frac{\nu_{1}^{2}}{2}-a_{1}(t)\zeta_{1}^{2}\nu_{1}]+\frac{D_{x}({B_{z}}^{2}+r_{1})}{{\zeta_{1}}^{2}}[b_{1}\left(t\right)\zeta_{1}\frac{\nu_{1}^{3}}{3}-a_{1}(t)\zeta_{1}^{2}\frac{\nu_{1}^{2}}{2}]],$$

(53) $$f^{st}\left(\zeta_{2},\nu_{2},t\right)=exp[\frac{D_{y}}{\zeta_{2}}[b_{2}\left(t\right)\zeta_{2}\frac{\nu_{2}^{2}}{2}-a_{2}(t)\zeta_{2}^{2}\nu_{2}]+\frac{D_{y}({B_{z}}^{2}+r_{2})}{{\zeta_{2}}^{2}}[b_{2}\left(t\right)\zeta_{2}\frac{\nu_{2}^{3}}{3}-a_{2}(t)\zeta_{2}^{2}\frac{\nu_{2}^{2}}{2}]].$$

The Fourier-space solutions can therefore be written as

(54) $$g\left(\zeta_{1},\nu_{1},t\right)=h(\zeta_{1},\nu_{1},t)exp[-D_{x}\int [b_{1}\left(t\right)\zeta_{1}\nu_{1}-a_{1}\left(t\right)\zeta_{1}^{2}]dt],$$

(55) $$g\left(\zeta_{2},\nu_{2},t\right)=h(\zeta_{2},\nu_{2},t)exp[-D_{y}\int \left[b_{2}\left(t\right)\zeta_{2}\nu_{2}-a_{2}\left(t\right)\zeta_{2}^{2}\right]dt].$$

In the long-time limit, the coefficients satisfy $a_{i}(t)=1$, $b_{i}(t)=-\tau$ and $\int a_{i}(t)dt=t-\tau$, $\int b_{i}(t)dt=-\tau t$ for i=1,2. Accordingly, the arbitrary functions become

(56) $$f\left(\zeta_{1},\nu_{1},t\right)=\Theta [t+\nu_{1}/[\zeta_{1}-D_{x}({B_{z}}^{2}+r_{1})\nu_{1}]]g_{\zeta_{1}}^{st}(\zeta_{1},\nu_{1},t)f^{st}(\zeta_{1},\nu_{1},t),$$

(57) $$f\left(\zeta_{2},\nu_{2},t\right)=\Theta [t+\nu_{2}/[\zeta_{2}-D_{x}({B_{z}}^{2}+r_{2})\nu_{2}]]g_{\zeta_{2}}^{st}(\zeta_{2},\nu_{2},t)f^{st}(\zeta_{2},\nu_{2},t).$$

After expanding in powers of t/τ and collecting all terms, we obtain

(58) $$f(\zeta_{1},\nu_{1},t)=exp[-\frac{D_{x}{B_{z}}^{2}t^{4}}{3}{\zeta_{1}}^{2}-\frac{D_{x}{B_{z}}^{4}t^{3}}{2}\nu_{1}^{2}],$$

(59) $$f(\zeta_{2},\nu_{2},t)=exp[-\frac{D_{y}{B_{z}}^{2}t^{4}}{3}{\zeta_{2}}^{2}-\frac{D_{y}{B_{z}}^{4}t^{3}}{2}\nu_{2}^{2}].$$

Taking the inverse Fourier transforms, we finally obtain

(60) $$f(x,t)=[4\pi D_{x}{{B_{z}}^{2}t}^{4}{/3]}^{-\frac{1}{2}}exp[-\frac{3x^{2}}{4D_{x}{B_{z}}^{2}t^{4}}],\;f\left(v_{x},t\right)=[2\pi D_{x}{B_{z}}^{4}t^{3}{]}^{-1/2}exp[-\frac{{v_{x}}^{2}}{2D_{x}{B_{z}}^{4}t^{3}}],$$

(61) $$f(y,t)=[4\pi D_{y}{{B_{z}}^{2}t}^{4}/3{]}^{-\frac{1}{2}}exp[-\frac{3y^{2}}{4D_{y}{B_{z}}^{2}t^{4}}],\;f(v_{y},t)=[2\pi D_{y}{B_{z}}^{4}t^{3}{]}^{-1/2}exp[-\frac{{v_{y}}^{2}}{2D_{y}{B_{z}}^{4}t^{3}}].$$

The mean-square displacement and velocity are therefore given by

(62) $$<x^{2}(t)>={2D}_{x}{B_{z}}^{2}t^{4}/3,\;<v_{x}^{2}(t)>=D_{x}{B_{z}}^{4}t^{3},$$

(63) $$<y^{2}(t)>={2D}_{y}{B_{z}}^{2}t^{4}/3,\;<v_{y}^{2}(t)>=D_{y}{B_{z}}^{4}t^{3}.$$

#### 3.1.3. f(x,t)*,* $f(v_{x},t)$ and f(y,t)*,* $f(v_{y},t)$ in the White-Noise Limit τ=0

For τ=0, the coefficients reduce to $a_{1}(t)=a_{2}(t)=1$ and $b_{1}(t)=b_{2}(t)=0$. Equations (32) and (33) then become

(64) $$\frac{\partial }{\partial t}f\left(\zeta_{1},\nu_{1},t\right)=\left[\zeta_{1}-D_{x}({B_{z}}^{2}+r_{1})\nu_{1}\right]\frac{\partial }{\partial \nu_{1}}f\left(\zeta_{1},\nu_{1},t\right)-D_{x}{\zeta_{1}}^{2}f(\zeta_{1},\nu_{1},t),$$

(65) $$\frac{\partial }{\partial t}f\left(\zeta_{2},\nu_{2},t\right)=\left[\zeta_{2}-D_{x}({B_{z}}^{2}+r_{2})\nu_{2}\right]\frac{\partial }{\partial \nu_{2}}f\left(\zeta_{2},\nu_{2},t\right)-D_{y}{\zeta_{2}}^{2}f(\zeta_{2},\nu_{2},t).$$

The steady-state solutions are

(66) $$f^{st}(\zeta_{1},\nu_{1},t)=exp[D_{x}\zeta_{1}\nu_{1}+\frac{D_{x}({B_{z}}^{2}+r_{1})}{2}\nu_{1}^{2}],$$

(67) $$f^{st}(\zeta_{2},\nu_{2},t)=exp[D_{y}\zeta_{2}\nu_{2}+\frac{D_{y}({B_{z}}^{2}+r_{2})}{2}\nu_{2}^{2}].$$

The corresponding Fourier-space solutions read

(68) $$f(\zeta_{1},\nu_{1},t)=\Theta [t+\nu_{1}/[\zeta_{1}-({B_{z}}^{2}+r_{1})\nu_{1}]]f^{st}(\zeta_{1},\nu_{1},t),$$

(69) $$f\left(\zeta_{2},\nu_{2},t\right)=\Theta [t+\nu_{2}/[\zeta_{2}-({B_{z}}^{2}+r_{2})\nu_{2}]]f^{st}(\zeta_{2},\nu_{2},t).$$

After evaluation, we obtain

(70) $$f(\zeta_{1},\nu_{1},t)=exp[-(D_{x}t+\frac{D_{x}{B_{z}}^{2}t^{2}}{2}{{)\zeta }_{1}}^{2}-\frac{D_{x}{B_{z}}^{4}t^{2}}{2}{\nu_{1}}^{2}],$$

(71) $$f\left(\zeta_{2},\nu_{2},t\right)=exp[-(D_{y}t+\frac{D_{y}{B_{z}}^{2}t^{2}}{2}{{)\zeta }_{2}}^{2}-\frac{D_{y}{B_{z}}^{4}t^{2}}{2}{\nu_{2}}^{2}].$$

For $D_{x}t\gg 1$ and $D_{y}t\gg 1$, the probability densities reduce to

(72) $$f(x,t)=[4\pi D_{x}t{]}^{-\frac{1}{2}}exp[-\frac{x^{2}}{4D_{x}t}],\;f\left(v_{x},t\right)=[2\pi D_{x}{B_{z}}^{4}t^{2}{]}^{-1/2}exp[-\frac{{v_{x}}^{2}}{2D_{x}{B_{z}}^{4}t^{2}}],$$

(73) $$f(y,t)=[4\pi D_{y}t{]}^{-\frac{1}{2}}exp[-\frac{y^{2}}{4D_{y}t}],\;f(v_{y},t)=[2\pi D_{y}{B_{z}}^{4}t^{2}{]}^{-1/2}exp[-\frac{{v_{y}}^{2}}{2D_{y}{B_{z}}^{4}t^{2}}].$$

The corresponding mean-square values are

(74) $$<x^{2}(t)>={2D}_{x}t,\;<v_{x}^{2}(t)>=D_{x}{B_{z}}^{4}t^{2},$$

(75) $$<y^{2}\left(t\right)>={2D}_{y}t,<v_{y}^{2}(t)>=D_{y}{B_{z}}^{4}t^{2}.$$

## 4. Vlasov Equation with Trap Forces, Thermal and Active Noises in the Magnetic Field

### 4.1. Vlasov Equation for a Charged Particle with Trap Forces and Thermal Noises

The thermal fractional Vlasov equations in a uniform magnetic field $\vec{B}=B_{z}\hat{z}$ are modified as follows:

(76) $$\frac{\partial }{\partial t}x=v_{x}+\xi_{x}^{th}\left(t\right),\;\frac{\partial }{\partial t}v_{x}=-k_{1}x-{B_{z}}^{2}v_{x}-r_{1}v_{x},$$

and

(77) $$\frac{\partial }{\partial t}y=v_{y}+\xi_{y}^{th}\left(t\right),\;\frac{\partial }{\partial t}v_{y}=-k_{2}y{{-B}_{z}}^{2}v_{y}-r_{2}v_{y}.$$

Here, the dimensionless parameters m=1 and q=1. The forces $-k_{1}x$ and $-k_{2}y$ represent harmonic trap forces. The thermal noises $\xi_{x}^{th}\left(t\right)$ and $\xi_{y}^{th}\left(t\right)$ are temporally correlated and satisfy $<\xi_{j}^{th}\left(t\right)\xi_{j}^{th}\left(t^{\prime }\right)>=D_{j}|\frac{t-t^{\prime }}{\tau_{th}}{|}^{2h-2}$ for j=x,y, where the persistent Hurst exponent *h* lies in the range 1/2<h<1. The parameter $\tau_{th}$ is the thermal correlation time for a charged particle subject to trap forces and the thermal noise. The joint probability densities $f(x,v_{x},t)$ and $f(y,v_{y},t)$ are defined by $f\left(x,v_{x},t\right)=<\delta \left(x-x\left(t\right)\right)\delta (v_{x}-v_{x}(t))>$ and $f(y,v_{y},t)=<\delta (y-y(t))\delta (v_{y}-v_{y}(t))>$. Taking time derivatives of the joint probability densities yields

(78) $$\frac{\partial }{\partial t}f\left(x,v_{x},t\right)=-\frac{\partial }{\partial x}<\frac{\partial x}{\partial t}\delta \left(x-x\left(t\right)\right)\delta \left(v_{x}-v_{x}\left(t\right)\right)>-\frac{\partial }{\partial v_{x}}<\frac{\partial v_{x}}{\partial t}\delta \left(x-x\left(t\right)\right)\delta \left(v_{x}-v_{x}\left(t\right)\right)>,$$

(79) $$\frac{\partial }{\partial t}f\left(y,v_{y},t\right)=-\frac{\partial }{\partial y}<\frac{\partial y}{\partial t}\delta \left(y-y\left(t\right)\right)\delta \left(v_{y}-v_{y}\left(t\right)\right)>-\frac{\partial }{\partial v_{y}}<\frac{\partial v_{y}}{\partial t}\delta \left(y-y\left(t\right)\right)\delta \left(v_{y}-v_{y}\left(t\right)\right).$$

Substituting Equation (76) into Equation (78) and Equation (77) into Equation (79), and following the procedure of Ref. [13], we obtain

(80) $$\frac{\partial }{\partial t}f\left(x,v_{x},t\right)=[-v_{x}\frac{\partial }{\partial x}+[k_{1}x+{{(B}_{z}}^{2}{+r_{1})v}_{x}]\frac{\partial }{\partial v_{x}}]f\left(x,v_{x},t\right)+D_{x}[\frac{t^{2h-1}}{\left(2h-1\right)\tau_{th}^{2h-1}}\frac{\partial^{2}}{\partial v_{x}\partial x}+\frac{t^{2h}}{2h\tau_{th}^{2h}}\frac{\partial^{2}}{\partial x^{2}}]f(x,v_{x},t),$$

(81) $$\frac{\partial }{\partial t}f\left(y,v_{y},t\right)={[-v}_{y}\frac{\partial }{\partial y}+[k_{2}y+{{(B}_{z}}^{2}{+r_{2})v}_{y}]\frac{\partial }{\partial v_{y}}]f\left(y{,v}_{y},t\right)+D_{y}[\frac{t^{2h-1}}{\left(2h-1\right)\tau_{th}^{2h-1}}\frac{\partial^{2}}{\partial v_{y}\partial y}+\frac{t^{2h}}{2h\tau_{th}^{2h}}\frac{\partial^{2}}{\partial y^{2}}]f\left(y,v_{y},t\right).$$

The initial conditions are $x_{0}=v_{x0}=0\;{and\;y}_{0}=v_{y0}=0$. Applying the double Fourier transform of Equations (9) and (10), we obtain

(82) $$\frac{\partial }{\partial t}f\left(\zeta_{1},\nu_{1},t\right)=-k_{1}\nu_{1}\frac{\partial }{\partial \zeta_{1}}+\left[\zeta_{1}-({B_{z}}^{2}+r_{1})\nu_{1}\right]\frac{\partial }{\partial \nu_{1}}f\left(\zeta_{1},\nu_{1},t\right)-D_{x}[\frac{t^{2h-1}}{\left(2h-1\right)\tau_{th}^{2h-1}}\zeta_{1}\nu_{1}+\frac{t^{2h}}{2h\tau_{th}^{2h}}\zeta_{1}^{2}]f(\zeta_{1},\nu_{1},t),$$

(83) $$\frac{\partial }{\partial t}f\left(\zeta_{2},\nu_{2},t\right)=-k_{2}\nu_{2}\frac{\partial }{\partial \zeta_{2}}+\left[\zeta_{2}-({B_{z}}^{2}+r_{2})\nu_{2}\right]\frac{\partial }{\partial \nu_{2}}f\left(\zeta_{2},\nu_{2},t\right)-D_{y}[\frac{t^{2h-1}}{\left(2h-1\right)\tau_{th}^{2h-1}}\zeta_{2}\nu_{2}+\frac{t^{2h}}{2h\tau_{th}^{2h}}\zeta_{2}^{2}]f(\zeta_{2},\nu_{2},t).$$

Finally, we determine the probability densities for a charged particle with trap forces and exponentially correlated Gaussian forces in the regimes $\;t\ll \tau_{th}$, $t\gg \tau_{th}$, and for $\tau_{th}=0$. The time domain $t\gg \tau_{th}$ is the one where the short-time singularity of the memory kernel averages out.

#### 4.1.1. f(x,t), $f(v_{x},t)$ and f(y,t), $f(v_{y},t)$ in $t\ll \tau_{th}$

Since $\frac{\partial }{\partial t}f\left(\zeta_{1},t\right)=0$ and $\frac{\partial }{\partial t}f\left(\nu_{1},t\right)=0$, the Fourier transform of the probability densities $f\left(\zeta_{1},t\right)$ and $f\left(\nu_{1},t\right)$ reach the steady states, denoted by $f^{st}\left(\zeta_{1},t\right)$ and $f^{st}\left(\nu_{1},t\right)$, respectively. Accordingly, the two steady-state equations can be written as

(84) $$-k_{1}\nu_{1}\frac{\partial }{\partial \zeta_{1}}f^{st}\left(\zeta_{1},t\right)-[\frac{D_{x}}{2}\left[\frac{t^{2h-1}}{\left(2h-1\right)\tau_{th}^{2h-1}}\zeta_{1}\nu_{1}+\frac{t^{2h}}{2h\tau_{th}^{2h}}\zeta_{1}^{2}\right]+C]f^{st}\left(\zeta_{1},t\right),$$

(85) $$\left[\zeta_{1}-({B_{z}}^{2}+r_{1})\nu_{1}\right]\frac{\partial }{\partial \nu_{1}}f^{st}\left(\nu_{1},t\right)-[\frac{D_{x}}{2}\left[\frac{t^{2h-1}}{\left(2h-1\right)\tau_{th}^{2h-2}}\zeta_{1}\nu_{1}+\frac{t^{2h}}{2h\tau_{th}^{2h-2}}\zeta_{1}^{2}\right]-C]f^{st}\left(\nu_{1},t\right).$$

Here, C is the separation constant. The Fourier transform of the probability density from Equation (84) is given by

(86) $$f^{st}\left(\zeta_{1},t\right)=exp[-\frac{D_{x}}{2k_{1}\nu_{1}}\left[\frac{t^{2h-1}}{\left(2h-1\right)\tau_{th}^{2h-1}}\frac{\zeta_{1}^{2}}{2}\nu_{1}+\frac{t^{2h}}{2h\tau_{th}^{2h}}\frac{\zeta_{1}^{3}}{3}\right]+\frac{C}{k_{1}\nu_{1}}\zeta_{1}].$$

To determine the Fourier transform of probability density for $\zeta_{1}$, we write $f\left(\zeta_{1},t\right)=g\left(\zeta_{1},t\right)f^{st}\left(\zeta_{1},t\right)$. Retaining terms up to order $t^{2}$/$\tau_{th}^{2}$, the functions $g\left(\zeta_{1},t\right)$ and $h\left(\zeta_{1},t\right)$ are obtained as

(87) $$g\left(\zeta_{1},t\right)=h\left(\zeta_{1},t\right)exp[\frac{D_{x}}{{2{(k}_{1}\nu_{1})}^{2}}\left[\frac{t^{2h-2}}{\tau_{th}^{2h-1}}\frac{\zeta_{1}^{3}}{6}\nu_{1}+\frac{t^{2h-1}}{\tau_{th}^{2h}}\frac{\zeta_{1}^{4}}{12}\right]],$$

(88) $$h\left(\zeta_{1},t\right)=p\left(\zeta_{1},t\right)exp\left[-\frac{D_{x}}{{2{(k}_{1}\nu_{1})}^{3}}\left[\frac{{(2h-1)t}^{2h-2}}{\tau_{th}^{2h}}\frac{\zeta_{1}^{5}}{60}\right]\right].$$

In Equation (88), the function $p(\zeta_{1},t)$ satisfies $\frac{\partial }{\partial t}p\left(\zeta_{1},t\right)=-k_{1}\nu_{1}\frac{\partial }{\partial \zeta_{1}}p(\zeta_{1},t)$. Since the Fourier transform of the probability density can be regarded as an arbitrary function of the variable $t-[\frac{\zeta_{1}}{k_{1}\nu_{1}}]$, $p\left(\zeta_{1},t\right)$, we obtain $p\left(\zeta_{1},t\right)=\Theta [t-[\frac{\zeta_{1}}{k_{1}\nu_{1}}]]$. Keeping terms up to order 1/$\tau_{th}^{3}$, the asymptotic form of the Fourier transform of the probability density becomes

(89) $$f\left(\zeta_{1},t\right)=p^{st}(\zeta_{1},t)h^{st}(\zeta_{1},t)g^{st}(\zeta_{1},t)f^{st}(\zeta_{1},t)=\Theta [t-[\frac{\zeta_{1}}{k_{1}\nu_{1}}]]h^{st}(\zeta_{1},t)g^{st}(\zeta_{1},t)f^{st}(\zeta_{1},t).$$

Following the same procedure, by using a similar procedure as in Equations (86)–(89), the Fourier transform $f(\nu_{1},t)$ obtained from Equation (85) is given by

(90) $$f\left(\nu_{1},t\right)=\Theta [t+\nu_{1}/[\zeta_{1}-({B_{z}}^{2}{+r_{1})\nu }_{1}]]h^{st}(\nu_{1},t)g^{st}(\nu_{1},t)f^{st}(\nu_{1},t).$$

Using Equations (89) and (90), the joint Fourier-space probability density is obtained as

(91) $$f(\zeta_{1},\nu_{1},t)=exp[-\frac{{3D}_{x}t^{2h+1}}{{4\tau }_{th}^{2h}}{\zeta_{1}}^{2}-\frac{D_{x}k_{1}^{2}t^{2h+3}}{{24\tau }_{th}^{2h}}{\nu_{1}}^{2}],$$

and, by an analogous procedure,

(92) $$f(\zeta_{2},\nu_{2},t)=exp[-\frac{{3D}_{y}t^{2h+1}}{{4\tau }_{th}^{2h}}{\zeta_{2}}^{2}-\frac{D_{y}k_{2}^{2}t^{2h+3}}{{24\tau }_{th}^{2h}}{\nu_{2}}^{2}].$$

By applying the inverse Fourier transform to Equations (91) and (92), the probability densities are obtained as

(93) $$f(x,t)=[\pi \frac{{3D}_{x}t^{2h+1}}{\tau_{th}^{2h}}{]}^{-\frac{1}{2}}exp[-\frac{\tau_{th}^{2h}x^{2}}{{3D}_{x}t^{2h+1}}],\;f\left(v_{x},t\right)=[\pi \frac{D_{y}k_{1}^{2}t^{2h+3}}{{6\tau }_{th}^{2h}}{]}^{-\frac{1}{2}}exp[-\frac{{6h\tau }_{th}^{2h}v_{x}^{2}}{D_{x}k_{1}^{2}t^{2h+3}}],$$

(94) $$f(y,t)=[\pi \frac{{3D}_{y}t^{2h+1}}{\tau_{th}^{2h}}{]}^{-\frac{1}{2}}exp[-\frac{\tau_{th}^{2h-1}y^{2}}{{3D}_{y}t^{2h+1}}],\;f\left(v_{y},t\right)=[\pi \frac{D_{y}k_{2}^{2}t^{2h+3}}{{6\tau }_{th}^{2h}}{]}^{-\frac{1}{2}}exp[-\frac{{6\tau }_{th}^{2h}v_{y}^{2}}{D_{y}k_{2}^{2}t^{2h+3}}]$$

#### 4.1.2. f(x,t) and $f(v_{x},t)$ in $t\gg \tau_{th}$

In this subsection, we derive the probability densities f(x,t) and $f(v_{x},t)$ in the long-time domain. From Equation (82), the approximate equations for $f(\zeta_{1},t)$ and $f(\nu_{1},t)$ in the limit $t\gg \tau_{th}$ can be written as

(95) $$\frac{\partial }{\partial t}f\left(\zeta_{1},t\right)≅-\frac{D_{x}}{2}[\frac{t^{2h-1}}{\left(2h-1\right)\tau_{th}^{2h-1}}\zeta_{1}\nu_{1}+\frac{t^{2h}}{2h\tau_{th}^{2h}}\zeta_{1}^{2}]f(\zeta_{1},t),$$

(96) $$\frac{\partial }{\partial t}f\left(\nu_{1},t\right)≅-\frac{D_{x}}{2}[\frac{t^{2h-1}}{\left(2h-1\right)\tau_{th}^{2h-1}}\zeta_{1}\nu_{1}+\frac{t^{2h}}{2h\tau_{th}^{2h}}\zeta_{1}^{2}]f(\nu_{1},t),$$

From these equations, we obtain

(97) $$f_{\zeta_{1}}\left(\zeta_{1},t\right)=exp[-\frac{D_{x}}{2}\int [\frac{t^{2h-1}}{\left(2h-1\right)\tau_{th}^{2h-1}}\zeta_{1}\nu_{1}+\frac{t^{2h}}{2h\tau_{th}^{2h}}\zeta_{1}^{2}]dt],$$

(98) $$f_{\nu_{1}}\left(\nu_{1},t\right)=exp[-\frac{D_{x}}{2}\int \left[\frac{t^{2h-1}}{\left(2h-1\right)\tau_{th}^{2h-1}}\zeta_{1}\nu_{1}+\frac{t^{2h}}{2h\tau_{th}^{2h}}\zeta_{1}^{2}]dt\right].$$

Writing $g_{\zeta_{1}}^{st}(\zeta_{1},t)$ and $g_{\nu_{1}}^{st}(\nu_{1},t)$ from ${f(\zeta_{1},\nu_{1},t)=g}_{\zeta_{1}}(\zeta_{1},\nu_{1},t)f_{\zeta_{1}}(\zeta_{1},\nu_{1},t)$, we obtain

(99) $$g_{\zeta_{1}}^{st}\left(\zeta_{1},t\right)=exp[\frac{D_{x}}{2}\int [\frac{t^{2h-1}}{\left(2h-1\right)\tau_{th}^{2h-1}}\zeta_{1}\nu_{1}+\frac{t^{2h}}{2h\tau_{th}^{2h}}\zeta_{1}^{2}]dt],$$

(100) $$g_{\nu_{1}}^{st}(\nu_{1},t)=exp[\frac{D_{x}}{2}\int [\frac{t^{2h-1}}{\left(2h-1\right)\tau_{th}^{2h-1}}\zeta_{1}\nu_{1}+\frac{t^{2h}}{2h\tau_{th}^{2h}}\zeta_{1}^{2}]dt].$$

The steady-state Fourier transforms obtained from Equation (86) are

(101) $$f^{st}\left(\zeta_{1},t\right)=exp[-\frac{D_{x}}{2k_{1}\nu_{1}}\left[\frac{t^{2h-1}}{\left(2h-1\right)\tau_{th}^{2h-1}}\frac{\zeta_{1}^{2}}{2}\nu_{1}+\frac{t^{2h}}{2h\tau_{th}^{2h}}\frac{\zeta_{1}^{3}}{3}\right]+\frac{C}{k_{1}\nu_{1}}\zeta_{1}],$$

and

(102) $$f^{st}\left(\nu_{1},t\right)=exp[\frac{D_{x}}{2\left[\zeta_{1}-({B_{z}}^{2}+r_{1})\nu_{1}\right]}\left[\frac{t^{2h-1}}{\left(2h-1\right)\tau_{th}^{2h-1}}\zeta_{1}\frac{\nu_{1}^{2}}{2}+\frac{t^{2h}}{2h\tau_{th}^{2h}}\zeta_{1}^{2}\nu_{1}\right]+\frac{C}{k_{1}\nu_{1}}\nu_{1}].$$

Thus

(103) $$g\left(\zeta_{1},t\right)=h(\zeta_{1},t)g_{\zeta_{1}}^{st}(\zeta_{1},t)=h(\zeta_{1},t)exp[\frac{D_{x}}{2}\int [\frac{t^{2h-1}}{\left(2h-1\right)\tau_{th}^{2h-1}}\zeta_{1}\nu_{1}+\frac{t^{2h}}{2h\tau_{th}^{2h}}\zeta_{1}^{2}]dt],$$

(104) $$g\left(\nu_{1},t\right)=h(\nu_{1},t)g_{\zeta_{2}}^{st}(\nu_{1},t)=h(\nu_{1},t)exp[\frac{D_{x}}{2}\int [\frac{t^{2h-1}}{\left(2h-1\right)\tau_{th}^{2h-1}}\zeta_{1}\nu_{1}+\frac{t^{2h}}{2h\tau_{th}^{2h}}\zeta_{1}^{2}]dt].$$

Taking the solutions as arbitrary functions of variables $t-\zeta_{1}/[k_{1}\nu_{1}]$ and $t+\nu_{1}/[\zeta_{1}-{B_{z}}^{2}\nu_{1}]$, we obtain $h(\zeta_{1},\nu_{1},t)=\Theta [t-\zeta_{1}/[k_{1}\nu_{1}]]$ and $h(\zeta_{2},\nu_{2},t)=\Theta [t+\nu_{1}/[\zeta_{1}-{B_{z}}^{2}\nu_{1}]]$. After expanding in powers of $t/\tau_{t}$ and performing cancellations, we obtain

(105) $$f\left(\zeta_{1},t\right)=\Theta [t-\zeta_{1}/[k_{1}\nu_{1}]]g_{\zeta_{1}}^{st}(\zeta_{1},t)f^{st}(\zeta_{1},t),$$

(106) $$f\left(\nu_{1},t\right)=\Theta [t+\nu_{1}/[\zeta_{1}-{{(B}_{z}}^{2}+r_{1})\nu_{1}]]g_{\nu_{1}}^{st}(\nu_{1},t)f^{st}(\nu_{1},t).$$

Hence,

(107) $$f(\zeta_{1},\nu_{1},t)=f(\zeta_{1},t)\;f(\nu_{1},t)=exp[-\frac{D_{x}t^{2h+1}}{{4h(2h+1)\tau }_{th}^{2h-1}}{\zeta_{1}}^{2}-\frac{D_{x}k_{1}^{2}t^{2h+3}}{{4h(2h+1)\tau }_{th}^{2h}}{\nu_{1}}^{2}].$$

Applying the inverse Fourier transform yields

(108) $$f(x,t)=[\pi \frac{D_{x}t^{2h+1}}{{h(2h+1)\tau }_{th}^{2h-1}}{]}^{-\frac{1}{2}}exp[-\frac{{h(2h+1)\tau }_{th}^{2h-1}x^{2}}{D_{x}t^{2h+1}}],\;f\left(v_{x},t\right)=[\pi \frac{D_{x}k_{1}^{2}t^{2h+3}}{{h(2h+1)\tau }_{th}^{2h}}{]}^{-\frac{1}{2}}exp[-\frac{{h(2h+1)\tau }_{th}^{2h}v_{x}^{2}}{D_{x}k_{1}^{2}t^{2h+3}}].$$

The mean squared displacement and velocity are therefore

(109) $$<x^{2}(t)>=\frac{D_{x}}{{2h(2h+1)\tau }_{th}^{2h-1}}t^{2h+1},\;<v_{x}^{2}(t)>=\frac{D_{x}k_{1}^{2}}{{2h(2h+1)\tau }_{th}^{2h}}t^{2h+3}.$$

We write approximate equations for the time derivatives of $f(\zeta_{2},t)$ and $f(\nu_{2},t)$ from Equation (83) in the long-time domain as

(110) $$\frac{\partial }{\partial t}f\left(\zeta_{2},t\right)≅\frac{D_{y}}{2}[\frac{t^{2h-1}}{\left(2h-1\right)\tau_{th}^{2h-2}}\zeta_{2}\nu_{2}+\frac{t^{2h}}{2h\tau_{th}^{2h-2}}\zeta_{2}^{2}]f(\zeta_{2},t),$$

(111) $$\frac{\partial }{\partial t}f\left(\nu_{2},t\right)≅\frac{D_{y}}{2}[\frac{t^{2h-1}}{\left(2h-1\right)\tau_{th}^{2h-2}}\zeta_{2}\nu_{2}+\frac{t^{2h}}{2h\tau_{th}^{2h-2}}\zeta_{2}^{2}]f(\nu_{2},t),$$

By using a similar procedure of Equations (97)–(106) for $\zeta_{2}\;and\;\nu_{2}$, we get the Fourier transforms of probability densities $f(\zeta_{2},t)$ and $f(\nu_{2},t)$ as

(112) $$f\left(\zeta_{2},t\right)=h\left(\zeta_{2},t\right)g_{\zeta_{1}}^{st}\left(\zeta_{2},t\right)f^{st}\left(\zeta_{2},t\right)=\Theta [t-\zeta_{2}/[k_{2}\nu_{2}]]g_{\zeta_{1}}^{st}\left(\zeta_{2},t\right)f^{st}\left(\zeta_{2},t\right),$$

(113) $$f\left(\nu_{2},t\right)=h(\nu_{2},t)g_{\nu_{2}}^{st}(\nu_{2},t)f^{st}(\nu_{2},t)=\Theta [t+\nu_{2}/[\zeta_{2}-{{(B}_{z}}^{2}+r_{2})\nu_{2}]]g_{\nu_{2}}^{st}(\nu_{2},t)f^{st}(\nu_{2},t).$$

From Equations (112) and (113), we calculate $f(\zeta_{2},\nu_{2},t)$ = $f(\zeta_{2},t)f(\nu_{2},t)$ as

(114) $$f(\zeta_{2},\nu_{2},t)=f(\zeta_{2},t)\;f(\nu_{2},t)=exp[-\frac{D_{x}t^{2h+1}}{{4h\left(2h+1\right)\tau }_{th}^{2h-1}}{\zeta_{2}}^{2}-\frac{D_{x}k_{1}^{2}t^{2h+3}}{{4h\left(2h+1\right)\tau }_{th}^{2h}}\nu_{2}^{2}].$$

We get the probability densities f(y,t) and $f(v_{y},t)$ from Equation (114) as

(115) $$f(y,t)=[\pi \frac{D_{x}t^{2h+1}}{{h\left(2h+1\right)\tau }_{th}^{2h-1}}{]}^{-\frac{1}{2}}exp[-\frac{{h\left(2h+1\right)\tau }_{th}^{2h-1}y^{2}}{D_{y}t^{2h+1}}],\;f\left(v_{y},t\right)=[\pi \frac{D_{y}k_{2}^{2}t^{2h+3}}{{h\left(2h+1\right)\tau }_{th}^{2h}}{]}^{-\frac{1}{2}}exp[-\frac{{h\left(2h+1\right)\tau }_{th}^{2h}v_{y}^{2}}{D_{y}k_{2}^{2}t^{2h+3}}],$$

with the mean squared displacement and the mean squared velocity for f(y,t) and $f(v_{y},t)$

(116) $$<y^{2}(t)>=\frac{D_{x}}{{2h\left(2h+1\right)\tau }_{th}^{2h-1}}t^{2h+1},\;<v_{y}^{2}(t)>=\frac{D_{y}k_{2}^{2}}{{2h\left(2h+1\right)\tau }_{th}^{2h}}t^{2h+3}.$$

#### 4.1.3. f(x,t), $f(v_{x},t)$ and f(y,t), $f(v_{y},t)$ in $\tau_{th}=0$

We write the approximate equation from Equation (82) for $\zeta_{1}$, $\nu_{1}$ and Equation (83) for $\zeta_{2}$, $\nu_{2}$ as

(117) $$\frac{\partial }{\partial t}f\left(\zeta_{1},t\right)≅-k_{1}\nu_{1}\frac{\partial }{\partial \zeta_{1}}f\left(\zeta_{1},t\right)-\frac{D_{x}}{2}[\frac{t^{2h}}{2h\tau_{th}^{2h-2}}\zeta_{1}^{2}]f(\zeta_{1},t),$$

(118) $$\frac{\partial }{\partial t}f\left(\nu_{1},t\right)≅\left[\zeta_{1}-({B_{z}}^{2}{+r_{1})\nu }_{1}\right]\frac{\partial }{\partial \nu_{1}}f\left(\nu_{1},t\right)-\frac{D_{x}}{2}[\frac{t^{2h}}{2h\tau_{th}^{2h}}\zeta_{1}^{2}]f(\nu_{1},t),$$

(119) $$\frac{\partial }{\partial t}f\left(\zeta_{2},t\right)≅-k_{2}\nu_{2}\frac{\partial }{\partial \zeta_{2}}f\left(\zeta_{2},t\right)-\frac{D_{y}}{2}[\frac{t^{2h}}{2h\tau_{th}^{2h}}\zeta_{2}^{2}]f(\zeta_{2},t),$$

(120) $$\frac{\partial }{\partial t}f\left(\nu_{2},t\right)≅\left[\zeta_{2}-{{(B}_{z}}^{2}{+r_{2})\nu }_{2}\right]\frac{\partial }{\partial \nu_{2}}f\left(\nu_{2},t\right)-\frac{D_{y}}{2}[\frac{t^{2h}}{2h\tau_{th}^{2h}}\zeta_{2}^{2}]f(\nu_{2},t).$$

From the above equations in the steady state, we calculate $f^{st}(\zeta_{1},t)$, $f^{st}(\nu_{1},t)$ and $f^{st}(\zeta_{2},t)$, $f^{st}(\nu_{2},t)$ as

(121) $$f^{st}\left(\zeta_{1}t\right)=exp[-\frac{D_{x}}{2k_{1}\nu_{1}}[\frac{t^{2h}}{2h\tau_{th}^{2h}}]\frac{{\zeta_{1}}^{3}}{3}],\;f^{st}(\nu_{1},t)=exp[\frac{D_{x}}{2\left[\zeta_{1}-{B_{z}}^{2}\nu_{1}\right]}[\frac{t^{2h}}{2h\tau_{th}^{2h}}]\zeta_{1}^{2}\nu_{1}],$$

(122) $$f^{st}(\zeta_{2},t)=exp[-\frac{D_{y}}{2k_{2}\nu_{2}}[\frac{t^{2h}}{2h\tau_{th}^{2h}}]\frac{{\zeta_{2}}^{3}}{3}],\;f^{st}(\nu_{2},t)=exp[\frac{D_{y}}{2\left[\zeta_{2}-{B_{z}}^{2}\nu_{2}\right]}[\frac{t^{2h}}{2h\tau_{th}^{2h}}]\zeta_{2}^{2}\nu_{2}].$$

The Fourier transforms of the probability densities $f\left(\zeta_{1},t\right),\;f(\nu_{1},t)$ and $f(\zeta_{2},t)$, $f(\nu_{2},t)$ are derived as

(123) $$f(\zeta_{1},t)=\Theta [t-\zeta_{1}/k_{1}\nu_{1}]f^{st}(\zeta_{1},t),\;f\left(\nu_{1},t\right)=\Theta [t+\nu_{1}/[\zeta_{1}-{{(B}_{z}}^{2}{+r_{1})\nu }_{1}]]f^{st}\left(\nu_{1},t\right),$$

(124) $$f(\zeta_{2},t)=\Theta [t-\zeta_{2}/k_{2}\nu_{2}]f^{st}(\zeta_{2},t),\;f\left(\nu_{2},t\right)=\Theta [t+\nu_{2}/[\zeta_{2}-({B_{z}}^{2}+r_{2})\nu_{2}]]f^{st}(\nu_{2},t).$$

We calculate $f\left(\zeta_{1},\nu_{1},t\right)$ and $f\left(\zeta_{2},\nu_{2},t\right)$ from Equations (123) and (124) as

(125) $$f\left(\zeta_{1},\nu_{1},t\right)=f(\zeta_{1},t)f(\nu_{1},t)=exp[-\frac{D_{x}t^{2h+1}}{2{h\tau }_{th}^{2h}}{\zeta_{1}}^{2}-\frac{D_{x}k_{1}^{2}t^{2h+3}}{{12h\tau }_{th}^{2h}}{\nu_{1}}^{2}],$$

(126) $$f\left(\zeta_{2},\nu_{2},t\right)=f(\zeta_{2},t)f\left(\nu_{2},t\right)=exp[-\frac{D_{y}t^{2h+1}}{2{h\tau }_{th}^{2h}}{\zeta_{2}}^{2}-\frac{D_{y}k_{2}^{2}t^{2h+3}}{{12h\tau }_{th}^{2h}}{\nu_{2}}^{2}].$$

By using the inverse Fourier transforms, f(x,t), $f(v_{x},t)$ and f(y,t), $f(v_{y},t)$ are, respectively, presented by

(127) $$f(x,t)=[2\pi \frac{D_{x}t^{2h+1}}{{h\tau }_{th}^{2h}}{]}^{-\frac{1}{2}}exp[-\frac{{h\tau }_{th}^{2h}x^{2}}{2D_{x}t^{2h+1}}],\;f\left(v_{x},t\right)=[\pi \frac{D_{x}k_{1}^{2}t^{2h+3}}{{3h\tau }_{th}^{2h}}{]}^{-\frac{1}{2}}exp[-\frac{{3h\tau }_{th}^{2h}v_{x}^{2}}{D_{x}k_{1}^{2}t^{2h+3}}],$$

(128) $$f(y,t)=[2\pi \frac{D_{y}{B_{z}^{2}t}^{2h+1}}{{h\tau }_{th}^{2h}}{]}^{-\frac{1}{2}}exp[-\frac{{h\tau }_{th}^{2h}y^{2}}{{2D}_{y}t^{2h+1}}],\;f\left(v_{y},t\right)=[\pi \frac{D_{y}k_{2}^{2}t^{2h+3}}{{3h\tau }_{th}^{2h}}{]}^{-\frac{1}{2}}exp[-\frac{{3h\tau }_{th}^{2h}v_{y}^{2}}{D_{y}k_{2}^{2}t^{2h+3}}].$$

The mean squared displacement and the mean squared velocity for f(x,t), $f(v_{x},t)$ and f(y,t), $f(v_{y},t)$ are, respectively, calculated as

(129) $$<x^{2}(t)>=\frac{D_{x}}{{h\tau }_{th}^{2h}}t^{2h+1},\;<v_{x}^{2}(t)>=\frac{D_{x}k_{1}^{2}}{{6h\tau }_{th}^{2h}}t^{2h+3},$$

(130) $$<y^{2}(t)>=\frac{D_{y}}{{h\tau }_{th}^{2h}}t^{2h+1},\;<v_{y}^{2}(t)>=\frac{D_{y}k_{2}^{2}}{{6h\tau }_{th}^{2h}}t^{2h+3}.$$

### 4.2. Vlasov Equation for a Charged Particle with Trap Forces and Active Noises

The Vlasov equations of motion for a charged particle with trap forces and active noises $\xi_{x}^{ac}\left(t\right)$, $\xi_{y}^{ac}\left(t\right)$ in the magnetic field $\vec{B}=B_{z}\hat{z}$ are given by

(131) $$\frac{\partial }{\partial t}x=v_{x}{+\xi }_{x}^{ac}\left(t\right),\;m\frac{\partial }{\partial t}v_{x}=-k_{1}x-{B_{z}}^{2}v_{x}-\gamma_{1}\int_{0}^{t}dt\prime |\frac{t-t^{\prime }}{\tau_{th}}{|}^{2h-2}v_{x}\left(t^{\prime }\right)-r_{1}v_{x},$$

and

(132) $$\frac{\partial }{\partial t}y=v_{y}{+\xi }_{y}^{ac}\left(t\right),\;m\frac{\partial }{\partial t}v_{y}=-k_{2}y{{-B}_{z}}^{2}v_{y}-\gamma_{2}\int_{0}^{t}dt\prime |\frac{t-t\prime }{\tau_{th}}{|}^{2h-2}v_{y}(t\prime )-r_{2}v_{y}.$$

Here, $-k_{1}x$ and $-k_{2}y$ denote the trap forces. The active noises $\xi_{x}^{ac}(t)$ and $\xi_{x}^{ac}(t)$ depends on the time difference: $<\xi_{j}^{ac}\left(t\right)\xi_{j}^{ac}\left(t^{\prime }\right)>=\frac{D_{i}}{\tau_{ac}}exp(-\frac{\left|t-t\prime \right|}{\tau_{ac}})$ for j=x,y and $\tau_{ac}$ is the active correlation time for a charged particle with the trap force and the active noise.

The joint probability densities $p(x,v_{x},t)$ and $p(y,v_{y},t)$ are defined by $f(x,v_{x},t)=<\delta (x-x(t))\delta (v_{x}-v_{x}(t))>$ and $f(y,v_{y},t)=<\delta (y-y(t))\delta (v_{y}-v_{y}(t))>$. By taking time derivatives of the joint probability density, we have the differential equations as

(133) $$\frac{\partial }{\partial t}f(x,v_{x},t)=-\frac{\partial }{\partial x}<\frac{\partial x}{\partial t}\delta (x-x(t))\delta (v_{x}-v_{x}(t))>-\frac{\partial }{\partial v_{x}}<\frac{\partial v_{x}}{\partial t}\delta (x-x(t))\delta (v_{x}-v_{x}(t))>,$$

(134) $$\frac{\partial }{\partial t}f\left(y,v_{y},t\right)=-\frac{\partial }{\partial y}<\frac{\partial y}{\partial t}\delta \left(y-y\left(t\right)\right)\delta \left(v_{y}-v_{y}\left(t\right)\right)>-\frac{\partial }{\partial v_{y}}<\frac{\partial v_{y}}{\partial t}\delta \left(y-y\left(t\right)\right)\delta \left(v_{y}-v_{y}\left(t\right)\right).$$

Inserting Equation (133) into Equation (135) and inserting Equation (134) into Equation (136), we have, following the method of Ref. [13],

(135) $$\frac{\partial }{\partial t}f\left(x,v_{x},t\right)=\left[-v_{x}\frac{\partial }{\partial x}+[k_{1}x+{{(B}_{z}}^{2}+r_{1})v_{x}]\frac{\partial }{\partial v_{x}}\right]f\left(x,v_{x},t\right)+D_{x}[{-b}_{1}\left(t\right)\frac{\partial^{2}}{\partial x^{2}}+a_{1}(t)\frac{\partial^{2}}{\partial \nu_{x}^{2}}]f(x,v_{x},t),$$

(136) $$\frac{\partial }{\partial t}f\left(y,v_{y},t\right)={[-v}_{y}\frac{\partial }{\partial y}+[k_{2}y+{{(B}_{z}}^{2}+r_{2})v_{y}]\frac{\partial }{\partial v_{y}}]f\left(y{,v}_{y},t\right)+D_{y}[{-b}_{2}\left(t\right)\frac{\partial^{2}}{\partial y^{2}}+a_{2}(t)\frac{\partial^{2}}{\partial \nu_{y}^{2}}]f\left(y,v_{y},t\right).$$

where the dimensionless parameters m=1 and q=1. The parameters $a_{1}(t)$, $a_{2}(t)$, $b_{1}(t)$, and $b_{2}(t)$ are, respectively, given by $a_{1}(t)=a_{2}(t)=1-exp(-t/\tau )$, and $b_{1}(t)=b_{2}(t)=(t+\tau )exp(-t/\tau )-\tau$. By using the double Fourier transform of Equations (9) and (10), we derive the Fourier transforms of Equations (135) and (136) as

(137) $$\frac{\partial }{\partial t}f\left(\zeta_{1},\nu_{1},t\right)=-k_{1}\nu_{1}\frac{\partial }{\partial \zeta_{1}}+\left[\zeta_{1}-{{(B}_{z}}^{2}+r_{1})\nu_{1}\right]\frac{\partial }{\partial \nu_{1}}f\left(\zeta_{1},\nu_{1},t\right)+D_{x}[b_{1}(t)\zeta_{1}\nu_{1}-a_{1}(t)\zeta_{1}^{2}]f(\zeta_{1},\nu_{1},t),$$

(138) $$\frac{\partial }{\partial t}f\left(\zeta_{2},\nu_{2},t\right)=-k_{2}\nu_{2}\frac{\partial }{\partial \zeta_{2}}+\left[\zeta_{2}-{{(B}_{z}}^{2}+r_{2})\nu_{2}\right]\frac{\partial }{\partial \nu_{2}}f\left(\zeta_{2},\nu_{2},t\right)+D_{y}[b_{2}(t)\zeta_{2}\nu_{2}-a_{2}(t)\zeta_{2}^{2}]f(\zeta_{2},\nu_{2},t).$$

Here, the initial condition is $x_{0}=v_{x0}=0,y_{0}=v_{y0}=0$.

Now, we find the Probability Densities for a charged particle with trap forces and exponential correlated Gaussian forces in $t\ll \tau_{ac}$, $t\gg \tau_{ac}$, and for $\tau_{ac}=0$. The limit domain $t\gg \tau_{ac}$ is the one where the active noise is reduced to effective white noise.

#### 4.2.1. f(x,t), $f(v_{x},t)$ and f(y,t), $f(v_{y},t)$ in $t<<\tau_{ac}$

As we have $\frac{\partial }{\partial t}f\left(\zeta_{1},t\right)=0$ and $\frac{\partial }{\partial t}f\left(\nu_{1},t\right)=0$, the Fourier transform of the probability density $f\left(\zeta_{1},t\right)$ and $f\left(\nu_{1},t\right)$ in the steady state becomes $f^{st}\left(\zeta_{1},t\right)$ and $f^{st}\left(\nu_{1},t\right)$. We write the two steady equations in the form:

(139) $$-k_{1}\nu_{1}\frac{\partial }{\partial \zeta_{1}}f^{st}\left(\zeta_{1},t\right)+[\frac{D_{x}}{2}\left[b_{1}\left(t\right)\zeta_{1}\nu_{1}-a_{1}\left(t\right)\zeta_{1}^{2}\right]+E]f^{st}\left(\zeta_{1},t\right),$$

(140) $$\left[\zeta_{1}-{{(B}_{z}}^{2}+r_{1})\nu_{1}\right]\frac{\partial }{\partial \nu_{1}}f^{st}\left(\nu_{1},t\right)+[\frac{D_{x}}{2}\left[b_{1}\left(t\right)\zeta_{1}\nu_{1}-a_{1}\left(t\right)\zeta_{1}^{2}\right]-E]f^{st}\left(\nu_{1},t\right).$$

Here, E is the separation constant. The Fourier transform of the probability density from Equation (139) is calculated as

(141) $$f^{st}\left(\zeta_{1},t\right)=exp[\frac{D_{x}}{2k_{1}\nu_{1}}\left[b_{1}\left(t\right)\frac{\zeta_{1}^{2}}{2}\nu_{1}-a_{1}\left(t\right)\frac{\zeta_{1}^{3}}{3}\right]+\frac{E}{k_{1}\nu_{1}}\zeta_{1}].$$

In order to find the Fourier transform of probability density for $\zeta_{1}$ from $f\left(\zeta_{1},t\right)=g\left(\zeta_{1},t\right)f^{st}\left(\zeta_{1},t\right)$, the Fourier transform of the probability density via the calculation including terms up to order $t^{2}$/$\tau^{2}$ are calculated as

(142) $$g\left(\zeta_{1},t\right)=h\left(\zeta_{1},t\right)exp[-\frac{D_{x}}{{2{(k}_{1}\nu_{1})}^{2}}\left[b_{1}^{\prime }\left(t\right)\frac{\zeta_{1}^{3}}{6}\nu_{1}-a_{1}^{\prime }\left(t\right)\frac{\zeta_{1}^{4}}{12}\right]],$$

(143) $$h(\zeta_{1},t)=p(\zeta_{1},t)exp[\frac{D_{x}}{{2{(k}_{1}\nu_{1})}^{3}}\left[b_{1}^{\prime \prime }\left(t\right)\frac{\zeta_{1}^{4}}{24}\nu_{1}-{a\prime }_{1}^{\prime }\left(t\right)\frac{\zeta_{1}^{5}}{60}\right]],$$

(144) $$p(\zeta_{1},t)=q(\zeta_{1},t)exp[-\frac{D_{x}}{{2{(k}_{1}\nu_{1})}^{4}}\left[b_{1}^{\prime \prime \prime }\left(t\right)\frac{\zeta_{1}^{6}}{120}\nu_{1}\right]].$$

In Equation (144), $q(\zeta_{1},t)$ obeys $\frac{\partial }{\partial t}q\left(\zeta_{1},t\right)=-k_{1}\nu_{1}\frac{\partial }{\partial \zeta_{1}}\;q(\zeta_{1},t)$. Using the Fourier transform of the probability density as an arbitrary function of the variable $t-[\frac{\zeta_{1}}{k_{1}\nu_{1}}]$, $q\left(\zeta_{1},t\right)$ is given by $q\left(\zeta_{1},t\right)=\Theta [t-[\frac{\zeta_{1}}{k_{1}\nu_{1}}]]$. The asymptotic Fourier transform of the probability density cut off in $q\left(\zeta_{1},t\right)$ up to the terms proportional to 1/$\tau_{ac}^{3}$. We consequently get $f\left(\zeta_{1},t\right)$ related to $q\left(\zeta_{1},t\right)$ as

(145) $$f\left(\zeta_{1},t\right)=q^{st}\left(\zeta_{1},t\right)p^{st}(\zeta_{1},t)h^{st}(\zeta_{1},t)g^{st}(\zeta_{1},t)f^{st}(\zeta_{1},t)=\Theta [t-[\frac{\zeta_{1}}{k_{1}\nu_{1}}]]p^{st}(\zeta_{1},t)h^{st}(\zeta_{1},t)g^{st}(\zeta_{1},t)f^{st}(\zeta_{1},t).$$

By using a similar procedure of Equations (141)–(145) for $\zeta_{1}$, $f(\nu_{1},t)$ is calculated as

(146) $$f\left(\nu_{1},t\right)=\Theta [t+\nu_{1}/[\zeta_{1}-{{(B}_{z}}^{2}+r_{1})\nu_{1}]]p^{st}(\nu_{1},t)h^{st}(\nu_{1},t)g^{st}(\nu_{1},t)f^{st}(\nu_{1},t).$$

we calculate $f\left(\zeta_{1},\nu_{1},t\right)=f(\zeta_{1},t)f(\nu_{1},t)$ from Equations (145) and (146) as

(147) $$f(\zeta_{1},\nu_{1},t)=exp[-\frac{D_{x}t^{3}}{4k_{1}}{\zeta_{1}}^{2}-\frac{{{D_{x}B}_{z}}^{4}t^{3}}{4k_{1}}{\nu_{1}}^{2}-\frac{D_{x}t^{3}}{4}{\zeta_{1}}^{2}-\frac{D_{x}{B_{z}}^{4}t^{3}}{4}\nu_{1}^{2}].$$

From Equation (149), using the inverse Fourier transform, the probability densities f(x,t) and $f(v_{x},t)$ are, respectively, presented by

(148) $$f(x,t)=[\pi D_{x}{k_{1}}^{-1}t^{3}{]}^{-\frac{1}{2}}exp[-\frac{k_{1}x^{2}}{D_{x}t^{3}}],\;f\left(v_{x},t\right)=[\pi D_{x}{B_{z}}^{4}{k_{1}}^{-1}t^{3}{]}^{-\frac{1}{2}}exp[-\frac{k_{1}x^{2}}{D_{x}{B_{z}}^{4}t^{3}}].$$

The mean squared displacement and the mean squared velocity for f(x,t) and $f(v_{x},t)$ are, respectively, given by

(149) $$<x^{2}(t)>=\frac{D_{x}}{2k_{1}}t^{3},\;<v_{x}^{2}(t)>=\frac{{{D_{x}B}_{z}}^{4}}{2k_{1}}t^{3}.$$

Using a similar procedure of Equations (141)–(149) for $\zeta_{2}$ and $\nu_{2}$, the probability densities f(y,t) and $f(v_{y},t)$ are, respectively, presented by

(150) $$f(y,t)=[\pi D_{y}{k_{2}}^{-1}t^{3}{]}^{-\frac{1}{2}}exp[-\frac{k_{2}y^{2}}{D_{y}t^{3}}],\;f\left(v_{y},t\right)=[\pi D_{y}{B_{z}}^{4}{k_{2}}^{-1}t^{3}{]}^{-\frac{1}{2}}exp[-\frac{k_{2}v_{y}^{2}}{D_{y}{B_{z}}^{4}t^{3}}].$$

The mean squared displacement and the mean squared velocity for f(x,t) and $f(v_{x},t)$ are, respectively, given by

(151) $$<y^{2}(t)>=\frac{D_{y}}{2k_{2}}t^{3},\;<v_{y}^{2}(t)>=\frac{{{D_{y}B}_{z}}^{4}}{2k_{2}}t^{3}.$$

#### 4.2.2. f(x,t) and $f(v_{x},t)$ in $t\gg \tau_{ac}$

In this subsection, we find the probability densities in the long-time domain $t\gg \tau_{ac}$. We can write approximate equations for $f(\zeta_{1},t)$ and $f(\nu_{1},t)$ from Equation (137) in the long-time domain as

(152) $$\frac{\partial }{\partial t}f\left(\zeta_{1},t\right)≅\frac{D_{x}}{2}[b_{1}(t)\zeta_{1}\nu_{1}-a_{1}(t)\zeta_{1}^{2}]f(\zeta_{1},t),$$

(153) $$\frac{\partial }{\partial t}f\left(\nu_{1},t\right)≅\frac{D_{x}}{2}[b_{1}(t)\zeta_{1}\nu_{1}-a_{1}(t)\zeta_{1}^{2}]f(\nu_{1},t),$$

From the above equations, we have

(154) $$f_{\zeta_{1}}\left(\zeta_{1},t\right)=exp[\frac{D_{x}}{2}\int [b_{1}(t)\zeta_{1}\nu_{1}-a_{1}(t)\zeta_{1}^{2}dt]],$$

(155) $$f_{\nu_{1}}\left(\nu_{1},t\right)=exp[\frac{D_{x}}{2}\int [b_{1}(t)\zeta_{1}\nu_{1}-a_{1}(t)\zeta_{1}^{2}dt]].$$

We get $g_{\zeta_{1}}^{st}(\zeta_{1},t)$ and $g_{\nu_{1}}^{st}(\nu_{1},t)$ from ${f(\zeta_{1},\nu_{1},t)=g}_{\zeta_{1}}(\zeta_{1},\nu_{1},t)f_{\zeta_{1}}(\zeta_{1},\nu_{1},t)$ and ${f(\nu_{1},t)=g}_{\nu_{1}}(\nu_{1},t)f_{\nu_{1}}(\nu_{1},t)$ as

(156) $$g_{\zeta_{1}}^{st}\left(\zeta_{1},t\right)=exp[-\frac{D_{x}}{2}\int [b_{1}(t)\zeta_{1}\nu_{1}-a_{1}(t)\zeta_{1}^{2}dt]],$$

(157) $$g_{\nu_{1}}^{st}(\nu_{1},t)=exp[-\frac{D_{x}}{2}\int [b_{1}(t)\zeta_{1}\nu_{1}-a_{1}(t)\zeta_{1}^{2}dt]].$$

$f^{st}\left(\zeta_{1},t\right)$ from Equation (141) is given by

(158) $$f^{st}\left(\zeta_{1},t\right)=exp[\frac{D_{x}}{2k_{1}\nu_{1}}\left[b_{1}\left(t\right)\frac{\zeta_{1}^{2}}{2}\nu_{1}-a_{1}\left(t\right)\frac{\zeta_{1}^{3}}{3}\right]+\frac{C}{k_{1}\nu_{1}}\zeta_{1}],$$

and $f^{st}(\nu_{1},t)$ is calculated as

(159) $$f^{st}\left(\nu_{1},t\right)=exp[\frac{D_{x}}{\zeta_{1}}[b_{1}\left(t\right)\zeta_{1}\frac{\nu_{1}^{2}}{2}-a_{1}(t)\zeta_{1}^{2}\nu_{1}]+\frac{{{D_{x}B}_{z}}^{2}}{{\zeta_{1}}^{2}}[b_{1}\left(t\right)\zeta_{1}\frac{\nu_{1}^{3}}{3}-a_{1}(t)\zeta_{1}^{2}\frac{\nu_{1}^{2}}{2}]].$$

The Fourier transforms of the probability densities $g(\zeta_{1},\nu_{1},t)$ and $g(\nu_{1},t)$ are given by

(160) $$g\left(\zeta_{1},t\right)=h(\zeta_{1},t)g_{\zeta_{1}}^{st}(\zeta_{1},t)=h(\zeta_{1},t)exp[-\frac{D_{x}}{2}\int [b_{1}\left(t\right)\zeta_{1}\nu_{1}-a_{1}\left(t\right)\zeta_{1}^{2}]dt],$$

(161) $$g\left(\nu_{1},t\right)=h(\nu_{1},t)g_{\zeta_{2}}^{st}(\nu_{1},t)=h(\nu_{1},t)exp[-\frac{D_{x}}{2}\int [b_{1}\left(t\right)\zeta_{1}\nu_{1}-a_{1}\left(t\right)\zeta_{1}^{2}]dt].$$

Here, $\int a_{i}\left(t\right)dt=t-\tau_{ac}$, $a_{i}(t)=1$ and $\int b_{i}\left(t\right)dt=-\tau_{ac}t$, $b_{i}\left(t\right)=-\tau_{ac}$ for i=1,2 in the long-time domain. Taking the solutions as arbitrary functions of variables $t-\zeta_{1}/[k_{1}\nu_{1}]$ and $t+\nu_{1}/[\zeta_{1}-{B_{z}}^{2}\nu_{1}]$, the arbitrary functions $h(\zeta_{1},\nu_{1},t)$ and $h(\zeta_{2},\nu_{2},t)$ become $h(\zeta_{1},\nu_{1},t)=\Theta [t-\zeta_{1}/[k_{1}\nu_{1}]]$ and $h(\zeta_{2},\nu_{2},t)=\Theta [t+\nu_{1}/[\zeta_{1}-{B_{z}}^{2}\nu_{1}]]$. By expanding in powers of $t/\tau_{t}$, we derive and calculate the expression for $f(\zeta_{1},t)$ and $f(\nu_{1},t)$ after some cancellations, as

(162) $$f\left(\zeta_{1},t\right)=h(\zeta_{1},t)g_{\zeta_{1}}^{st}(\zeta_{1},t)f^{st}(\zeta_{1},t)=\Theta [t-\zeta_{1}/[k_{1}\nu_{1}]]g_{\zeta_{1}}^{st}(\zeta_{1},t)f^{st}(\zeta_{1},t),$$

(163) $$f\left(\nu_{1},t\right)=h(\nu_{1},t)g_{\nu_{1}}^{st}(\nu_{1},t)f^{st}(\nu_{1},t)=\Theta [t+\nu_{1}/[\zeta_{1}-{{(B}_{z}}^{2}+r_{1})\nu_{1}]]g_{\nu_{1}}^{st}(\nu_{1},t)f^{st}(\nu_{1},t).$$

Therefore, from Equations (162) and (163), we calculate $f(\zeta_{1},\nu_{1},t)$ = $f(\zeta_{1},t)\;f(\nu_{1},t)$ as

(164) $$f(\zeta_{1},\nu_{1},t)=f(\zeta_{1},t)\;f(\nu_{1},t)=exp[-\frac{D_{x}t^{3}}{4k_{1}}{\zeta_{1}}^{2}-\frac{D_{x}{B_{z}}^{4}t^{3}}{4k_{1}}\nu_{1}^{2}-\frac{D_{x}t^{3}}{4}{\zeta_{1}}^{2}-\frac{D_{x}{B_{z}}^{4}t^{3}}{4}\nu_{1}^{2}].$$

From Equation (164), using the inverse Fourier transform, the probability densities f(x,t) and $f(v_{x},t)$ are, respectively, presented by

(165) $$f(x,t)=[\pi D_{x}k_{1}^{-1}t^{3}{]}^{-\frac{1}{2}}exp[-\frac{k_{1}x^{2}}{D_{x}t^{3}}],\;f\left(v_{x},t\right)=[\pi D_{x}{B_{z}}^{4}k_{1}^{-1}t^{3}{]}^{-\frac{1}{2}}exp[-\frac{k_{1}v_{x}^{2}}{D_{x}{B_{z}}^{4}t^{3}}].$$

The mean squared displacement and the mean squared velocity for f(x,t) and $f(v_{x},t)$ are, respectively, given by

(166) $$<x^{2}(t)>=\frac{D_{x}}{2k_{1}}t^{3},\;<v_{x}^{2}(t)>=\frac{{{D_{x}B}_{z}}^{4}}{2k_{1}}t^{3}.$$

In $t\gg \tau_{ac}$, we write approximate equations for the time derivatives of $f(\zeta_{2},t)$ and $f(\nu_{2},t)$ from Equation (138) in the long-time domain as

(167) $$\frac{\partial }{\partial t}f\left(\zeta_{2},t\right)≅\frac{D_{y}}{2}[b_{2}(t)\zeta_{2}\nu_{2}-a_{2}(t)\zeta_{2}^{2}]f(\zeta_{2},t),$$

(168) $$\frac{\partial }{\partial t}f\left(\nu_{2},t\right)≅\frac{D_{y}}{2}[b_{2}(t)\zeta_{2}\nu_{2}-a_{2}(t)\zeta_{2}^{2}]f(\nu_{2},t),$$

By using a similar procedure of Equations (154)–(164) for $\zeta_{2}\;and\;\nu_{2}$, we get the Fourier transforms of probability densities $f(\zeta_{2},t)$ and $f(\nu_{2},t)$ as

(169) $$f\left(\zeta_{2},t\right)=h\left(\zeta_{2},t\right)g_{\zeta_{1}}^{st}\left(\zeta_{2},t\right)f^{st}\left(\zeta_{2},t\right)=\Theta [t-\zeta_{2}/[k_{2}\nu_{2}]]g_{\zeta_{1}}^{st}\left(\zeta_{2},t\right)f^{st}\left(\zeta_{2},t\right),$$

(170) $$f\left(\nu_{2},t\right)=h(\nu_{2},t)g_{\nu_{2}}^{st}(\nu_{2},t)f^{st}(\nu_{2},t)=\Theta [t+\nu_{2}/[\zeta_{2}-{{(B}_{z}}^{2}+r_{2})\nu_{2}]]g_{\nu_{2}}^{st}(\nu_{2},t)f^{st}(\nu_{2},t).$$

From Equations (169) and (170), we calculate $f\left(\zeta_{2},\nu_{2},t\right)$ = $f(\zeta_{2},t)f(\nu_{2},t)$ as

(171) $$f\left(\zeta_{2},\nu_{2},t\right)\;=f(\zeta_{2},t)\;f(\nu_{2},t)=exp[-\frac{D_{y}t^{3}}{4k_{2}}{\zeta_{2}}^{2}-\frac{D_{y}{B_{z}}^{4}t^{3}}{4k_{2}}\nu_{2}^{2}-\frac{D_{y}t^{3}}{4}{\zeta_{2}}^{2}-\frac{D_{y}{B_{z}}^{4}t^{3}}{4}\nu_{2}^{2}].$$

We get the probability densities f(y,t) and $f(v_{y},t)$ from Equation (171) as

(172) $$f(y,t)=[\pi D_{y}k_{2}^{-1}t^{3}{]}^{-\frac{1}{2}}exp[-\frac{k_{2}y^{2}}{D_{y}t^{3}}],\;f\left(v_{y},t\right)=[\pi D_{y}{B_{z}}^{4}k_{2}^{-1}t^{3}{]}^{-\frac{1}{2}}exp[-\frac{k_{2}v_{y}^{2}}{D_{y}{B_{z}}^{4}t^{3}}],$$

with the mean squared displacement and the mean squared velocity for f(y,t) and $f(v_{y},t)$

(173) $$<y^{2}(t)>=\frac{D_{y}}{2k_{2}}t^{3},\;<v_{y}^{2}(t)>=\frac{{{D_{y}B}_{z}}^{4}}{2k_{2}}t^{3}.$$

#### 4.2.3. f(x,t), $f(v_{x},t)$ and f(y,t), $f(v_{y},t)$ in $\tau_{ac}=0$

For $\tau_{ac}=0$ ($a_{1}\left(t\right)=a_{2}(t)=1$ and $b_{1}\left(t\right)=b_{2}(t)=0$), we write the approximate equation from Equation (137) for $\zeta_{1}$, $\nu_{1}$ and Equation (138) for $\zeta_{2}$, $\nu_{2}$ as

(174) $$\frac{\partial }{\partial t}f\left(\zeta_{1},t\right)≅-k_{1}\nu_{1}\frac{\partial }{\partial \zeta_{1}}f\left(\zeta_{1},t\right)-\frac{D_{x}}{2}\zeta_{1}^{2}f(\zeta_{1},t),$$

(175) $$\frac{\partial }{\partial t}f\left(\nu_{1},t\right)≅\left[\zeta_{1}-{{(B}_{z}}^{2}+r_{1})\nu_{1}\right]\frac{\partial }{\partial \nu_{1}}f\left(\nu_{1},t\right)-\frac{D_{x}}{2}\zeta_{1}^{2}f(\nu_{1},t),$$

(176) $$\frac{\partial }{\partial t}f\left(\zeta_{2},t\right)≅-k_{2}\nu_{2}\frac{\partial }{\partial \zeta_{2}}f\left(\zeta_{2},t\right)-\frac{D_{y}}{2}\zeta_{2}^{2}f(\zeta_{2},t),$$

(177) $$\frac{\partial }{\partial t}f\left(\nu_{2},t\right)≅\left[\zeta_{2}-{{(B}_{z}}^{2}+r_{2})\nu_{2}\right]\frac{\partial }{\partial \nu_{2}}f\left(\nu_{2},t\right)-\frac{D_{y}}{2}\zeta_{2}^{2}f(\nu_{2},t).$$

From the above equations in the steady state, we calculate $f^{st}(\zeta_{1},t)$, $f^{st}(\nu_{1},t)$ and $f^{st}(\zeta_{2},t)$, $f^{st}(\nu_{2},t)$ as

(178) $$f^{st}\left(\zeta_{1}t\right)=exp[-\frac{D_{x}}{2k_{1}\nu_{1}}\zeta_{1}\nu_{1}^{2}],\;f^{st}(\nu_{1},t)=exp[\frac{D_{x}}{2\left[\zeta_{1}-{B_{z}}^{2}\nu_{1}\right]}\frac{{\nu_{1}}^{3}}{3}],$$

(179) $$f^{st}(\zeta_{2},t)=exp[-\frac{D_{y}}{2k_{2}\nu_{2}}\zeta_{2}\nu_{2}^{2}],\;f^{st}(\nu_{2},t)=exp[\frac{D_{y}}{2\left[\zeta_{2}-{B_{z}}^{2}\nu_{2}\right]}\frac{{\nu_{2}}^{3}}{3}].$$

The Fourier transforms of the probability densities $f\left(\zeta_{1},t\right),\;f(\nu_{1},t)$ and $f(\zeta_{2},t)$, $f(\nu_{2},t)$ are derived as

(180) $$f(\zeta_{1},t)=\Theta [t-\zeta_{1}/k_{1}\nu_{1}]f^{st}(\zeta_{1},t),\;f\left(\nu_{1},t\right)=\Theta [t+\nu_{1}/[\zeta_{1}-{{(B}_{z}}^{2}+r_{1})\nu_{1}]]f^{st}\left(\nu_{1},t\right),$$

(181) $$f(\zeta_{2},t)=\Theta [t-\zeta_{2}/k_{2}\nu_{2}]f^{st}(\zeta_{2},t),\;f\left(\nu_{2},t\right)=\Theta [t+\nu_{2}/[\zeta_{2}-{{(B}_{z}}^{2}+r_{2})\nu_{2}]]f^{st}(\nu_{2},t).$$

We calculate $f\left(\zeta_{1},\nu_{1},t\right)$ and $f\left(\zeta_{2},\nu_{2},t\right)$ from Equations (180) and (181) as

(182) $$f\left(\zeta_{1},\nu_{1},t\right)=f(\zeta_{1},t)f(\nu_{1},t)=exp[-\frac{D_{x}t^{3}}{6}{\zeta_{1}}^{2}-\frac{D_{x}t}{2}{\nu_{1}}^{2}],$$

(183) $$f\left(\zeta_{2},\nu_{2},t\right)=f(\zeta_{2},t)f\left(\nu_{2},t\right)=exp[-\frac{D_{y}t^{3}}{6}{\zeta_{2}}^{2}-\frac{D_{y}t}{2}{\nu_{2}}^{2}].$$

By using the inverse Fourier transforms, f(x,t), $f(v_{x},t)$ and f(y,t), $f(v_{y},t)$ are, respectively, presented by

(184) $$f(x,t)=[2\pi \frac{D_{x}t^{3}}{3}{]}^{-\frac{1}{2}}exp[-\frac{3x^{2}}{2D_{x}t^{3}}],\;f(v_{x},t)=[2\pi D_{x}t{]}^{-1/2}exp[-\frac{{v_{x}}^{2}}{2D_{x}t}]$$

(185) $$f(y,t)=[2\pi \frac{D_{y}t^{3}}{3}{]}^{-\frac{1}{2}}exp[-\frac{3y^{2}}{{2D}_{y}t^{3}}],\;f(v_{y},t)=[4\pi D_{y}t{]}^{-1/2}exp[-\frac{{v_{y}}^{2}}{2D_{y}t}].$$

The mean squared displacement and the mean squared velocity for f(x,t), $f(v_{x},t)$ and f(y,t), $f(v_{y},t)$ are, respectively, calculated as

(186) $$<x^{2}(t)>=\frac{D_{x}}{3}t^{3},\;<v_{x}^{2}(t)>=D_{x}t,$$

(187) $$<y^{2}(t)>=\frac{D_{y}}{3}t^{3},<v_{y}^{2}(t)>=D_{y}t.$$

## 5. Statistical Quantities and Moments

In this section, we calculate the non-Gaussian parameter, the correlation coefficient, the entropy, and the combined entropy for the displacement and the velocity. First of all, the non-Gaussian parameters for displacement and velocity are, respectively, given by

(188) $$K_{x}\;=\;<x^{4}>/{3<x^{2}>}^{2},\;K_{v_{x}}=<v_{x}^{4}>/{3<v_{x}^{2}>}^{2},$$

(189) $$K_{y}\;=\;<y^{4}>/{3<y^{2}>}^{2},K_{v_{y}}=<v_{y}^{4}>/{3<v_{y}^{2}>}^{2}.$$

Non-normal distribution parameters are expressed as the tails of the probability distribution function of a real variable, providing readers with insights into specific characteristics of probability distributions in probability theory and statistics. We introduce the correlation coefficient as

(190) $$\rho_{x,v_{x}}{=x}_{0}v_{x0}/\sigma_{x}\sigma_{v_{x}},\rho_{y,v_{y}}{=y}_{0}v_{y0}/\sigma_{y}\sigma_{v_{y}}.$$

Correlation coefficient $\rho_{x,v_{x}}$ means the statistical quantity describing the strength and direction of a relationship between two variables x and $v_{x}$. In the initial conditions, we assume that a passive particle is initially at $x\;{=\;x}_{0}$ and at ${v_{x}=v}_{x0}$. $\sigma_{x}$ and $\sigma_{v_{x}}$ denote the root-mean-squared displacement and the root-mean-squared velocity of the joint probability density. The entropies $S\left(x,t\right)$, $S\left(v_{x},t\right)$ and $S\left(y,t\right)$, $S\left(v_{y},t\right)$ are, respectively, calculated as

(191) $$S\left(x,t\right)=-\int dxf(x,t)lnf(x,t),S\left(v_{x},t\right)=-\int dv_{x}f(v_{x},t)lnf(v_{x},t),$$

(192) $$S\left(y,t\right)=-\int dyf(y,t)lnf(y,t),S\left(v_{y},t\right)=-\int dv_{y}f(v_{y},t)lnf(v_{y},t).$$

and the combined entropy is defined by

(193) $$S\left(x,v_{x},t\right)=-\int dx\int dv_{x}f(x,v_{x},t)lnf(x,v_{x},t),S\left(y,v_{y},t\right)=-\int dy\int dv_{y}f(y,v_{y},t)lnf(y,v_{y},t).$$

The entropy simply refers to a numerical representation of the reliability or quantity of information that has a probability distribution; in a probability distribution, as the probability of a particular value increases and the probability of the remaining values decreases, the entropy decreases.

In multiplying and integrating both sides of the Fokker–Plank equations by $x^{m}v^{n}$, the moment equations for a charged particle from Equations (7) and (8) are expressed as

(194) $$\frac{d}{dt}\mu_{m,n}=-m\mu_{m-1,n+1}+B_{z}v_{y0}n\mu_{m,n-1}+{B_{z}}^{2}n\mu_{m,n}+D_{x}n(n-1)\mu_{m-2,n},$$

(195) $$\frac{d}{dt}{\bar{\mu }}_{m,n}=-m{\bar{\mu }}_{m-1,n+1}-B_{z}v_{x0}n{\bar{\mu }}_{m,n-1}+{B_{z}}^{2}n{\bar{\mu }}_{m,n}+D_{x}n(n-1){\bar{\mu }}_{m-2,n}.$$

Here, $\mu_{m,n}=\int_{-\infty }^{+\infty }dx\int_{-\infty }^{+\infty }dv_{x}x^{m}v_{x}^{n}p\left(x,v_{x},t\right),$ and ${\bar{\mu }}_{m,n}=\int_{-\infty }^{+\infty }dy\int_{-\infty }^{+\infty }dv_{y}y^{m}v_{y}^{n}p\left(y,v_{y},t\right).$ The moment equations from Equations (30) and (31) for a charged particle with correlated Gaussian forces have

(196) $$\frac{d}{dt}\mu_{m,n}=-m\mu_{m-1,n+1}+B_{z}v_{y0}n\mu_{m,n-1}+{{(B}_{z}}^{2}+r_{1})n\mu_{m,n-1}+D_{x}[-mnb_{1}(t)\mu_{m-1,n-1}+m(m-1)a_{1}(t)\mu_{m-2,n}],$$

(197) $$\frac{d}{dt}{\bar{\mu }}_{m,n}=-m{\bar{\mu }}_{m-1,n+1}+-B_{z}v_{x0}n{\bar{\mu }}_{m,n-1}+{{(B}_{z}}^{2}+r_{2})n{\bar{\mu }}_{m,n-1}+D_{y}[-mnb_{2}(t){\bar{\mu }}_{m-1,n-1}+m(m-1)a_{2}(t){\bar{\mu }}_{m-2,n}].$$

For the thermal noise, the moment equations in Equations (80) and (81) are given by

(198) $$\frac{d}{dt}\mu_{m,n}=-m\mu_{m-1,n+1}+k_{1}\mu_{m+1,n}+{{(B}_{z}}^{2}+r_{2})n\mu_{m,n-1}+D_{x}[mn\frac{t^{2h-1}}{\left(2h-1\right)\tau_{th}^{2h-2}}\mu_{m-1,n-1}+m(m-1)\frac{t^{2h}}{2h\tau_{th}^{2h-2}}\mu_{m-2,n}],$$

(199) $$\frac{d}{dt}{\bar{\mu }}_{m,n}=-m{\bar{\mu }}_{m-1,n+1}+k_{2}{\bar{\mu }}_{m+1,n}+{{(B}_{z}}^{2}+r_{2})n{\bar{\mu }}_{m,n-1}+D_{y}[mn\frac{t^{2h-1}}{\left(2h-1\right)\tau_{th}^{2h-2}}{\bar{\mu }}_{m-1,n-1}+m(m-1)\frac{t^{2h}}{2h\tau_{th}^{2h-2}}{\bar{\mu }}_{m-2,n}].$$

For the active noise, the moment equations from Equations (135) and (136) are given by

(200) $$\frac{d}{dt}\mu_{m,n}=-m\mu_{m-1,n+1}+k_{1}\mu_{m+1,n}+{{(B}_{z}}^{2}+r_{1})n\mu_{m,n-1}+D_{x}[-mnb_{1}(t)\mu_{m-1,n-1}+m(m-1)a_{1}(t)\mu_{m-2,n}],$$

(201) $$\frac{d}{dt}{\bar{\mu }}_{m,n}=-m{\bar{\mu }}_{m-1,n+1}+k_{2}{\bar{\mu }}_{m+1,n}+{{(B}_{z}}^{2}+r_{2})n{\bar{\mu }}_{m,n-1}+D_{y}[-mnb_{2}(t){\bar{\mu }}_{m-1,n-1}+m(m-1)a_{2}(t){\bar{\mu }}_{m-2,n}].$$

Table 1 shows the non-Gaussian parameter, correlation coefficient, entropy, combined entropy, and moment for the motion of a charged particle in the short- and long-time domains, and Table 2 these values of statistical quantities for the motion of a charged particle with two correlated Gaussian forces. Table 3 and Table 4, respectively, summarize the values of the non-Gaussian parameter, correlation coefficient, entropy, combined entropy, and moment for the motion of a charged particle with the trap forces and the thermal noises, the trap forces and the active noises within the limits of three-times.

## 6. Conclusions

In this work, the double Fourier transform method has been applied to the two-dimensional Vlasov equation describing charged particles subjected to white noise, correlated Gaussian forces, trap forces, and thermal and active noises in a magnetic field. By deriving the associated Fokker–Planck equation, analytical solutions for the joint probability density in different time regimes were obtained. The inclusion of a viscous term suppresses persistent cyclotron oscillations and guarantees the existence of stationary probability densities in the long-time limit. In the long-time regime (t≫τ), exponentially correlated forces effectively reduce to white noise, and the particle dynamics approach an Ornstein–Uhlenbeck process in a magnetic field. In this limit, the viscous coefficient explicitly determines both the stationary velocity variance and the effective diffusion coefficient, ensuring convergence of the mean squared displacement.

The main results can be summarized as follows:

(1) In the short-time regime (t≪τ), where force correlations persist, particle motion resembles ballistic behavior rather than ordinary diffusion, since the random forces are not yet fully decorrelated. In this regime, the mean squared displacement scales as $\sim t^{2}$, reflecting an approximately constant velocity. In the long-time domain when $1\gg {B_{z}}^{2}t$, the mean squared displacement and velocity are given by $<x^{2}\left(t\right)>\;\sim \;t$ and $<v_{x}^{2}\left(t\right)>$ $\sim \;t^{2}$, respectively.
(2) In contrast to ordinary Brownian motion, where the mean squared displacement grows linearly with time, the Lorentz force does not significantly suppress diffusion in the short-time limit. However, in the long-time limit, regular diffusive behavior is recovered, with a diffusion coefficient explicitly determined by viscous damping. This demonstrates that the scaling behavior of charged particle motion in a magnetic field depends sensitively on the interplay between magnetic confinement and stochastic or collisional effects.
(3) For charged particles driven by correlated Gaussian noise, the mean squared displacement grows as $\langle x^{2}(t)\rangle ∼t^{4}$, indicating a ballistic-like superdiffusive behavior arising from the time-integrated acceleration induced by Lorentz-force–coupled correlated noise.

From the derived Fokker–Planck equation, we obtained approximate analytical expressions for the joint probability density [9,10,11], which were further validated through numerical simulations based on generalized Langevin equations describing both passive and active particles. These results provide a theoretical framework that can be tested experimentally in future studies of passive and active systems. Extensions of the present model to generalized Langevin equations or equations of motion involving additional types of forces are expected to yield further insights into stochastic transport properties in complex active systems [15,16,17] and to enable direct comparisons [18,19,20] with theoretical [21,22,23], numerical, experimental and other published investigations [24,25,26,27,28].

## Figures and Tables

**Table 1:** Values of the non-Gaussian parameter, the correlation coefficient, the entropy, the combined entropy, and the moments for the motion of a charged particle with $\vec{B}=B_{z}\vec{z}$ in the short- and long-time domains. Here, we assume that a charged particle is initially at $x=x_{0}$, $v_{x}=v_{x0}$ and at ${y=y}_{0}$, $v_{y}=v_{y0}$.

Time	${x,v}_{x}$ ${y,v}_{y}$	${K_{x},K}_{v_{x}}$ ${K_{y},K}_{v_{y}}$	$\rho_{x,v_{x}}$, $\rho_{{y,v}_{y}}$	$S\left(x,t\right),S(v_{x},t)$ $S\left(y,t\right),S(v_{y},t)$	$S({x,v}_{x},t)$ $S({y,v}_{y},t)$	$\mu_{2,2}$, ${\bar{\mu }}_{2,2}$
short- time	x	$\frac{x_{0}^{4}}{{D_{x}^{2}B}_{z}^{4}}t^{-4}+\frac{x_{0}^{2}}{D_{x}B_{z}^{2}}t^{-2}$	$\frac{x_{0}v_{x0}}{{D_{x}B}_{z}^{4}}t^{-2}$	${lnD}_{x}B_{z}^{2}t^{2}$	$lnD_{x}^{2}B_{z}^{8}t^{4}$	$\frac{{2D}_{x}^{2}B_{z}^{6}}{3}t^{3}$
	$v_{x}$	$\frac{v_{x0}^{4}}{{D_{x}^{2}B}_{z}^{12}}t^{-4}+\frac{v_{x0}^{2}}{D_{x}B_{z}^{6}}t^{-2}$		${lnD}_{x}B_{z}^{6}t^{2}$		
	y	$\frac{y_{0}^{4}}{{D_{y}^{2}B}_{z}^{4}}t^{-4}+\frac{y_{0}^{2}}{D_{y}B_{z}^{2}}t^{-2}$	$\frac{y_{0}v_{y0}}{{D_{y}B}_{z}^{4}}t^{-2}$	${lnD}_{y}B_{z}^{2}t^{2}$	$lnD_{y}^{2}B_{z}^{8}t^{4}$	$\frac{2D_{y}^{2}B_{z}^{6}}{3}t^{3}$
	$v_{y}$	$\frac{v_{y0}^{4}}{{D_{y}^{2}B}_{z}^{12}}t^{-4}+\frac{v_{y0}^{2}}{D_{y}B_{z}^{6}}t^{-2}$		${lnD}_{y}B_{z}^{6}t^{2}$		
long- time	x	$\frac{x_{0}^{4}}{D_{x}^{2}}t^{-2}+\frac{x_{0}^{2}}{D_{x}}t^{-1}$	$\frac{x_{0}v_{x0}}{{D_{x}B}_{z}^{3}}t^{-3/2}$	${lnD}_{x}t$	$lnD_{x}^{2}B_{z}^{7}t^{3}$	$\frac{{2D}_{x}^{2}B_{z}^{6}}{3}t^{3}$
	$v_{x}$	$\frac{v_{x0}^{4}}{{D_{x}^{2}B}_{z}^{12}}t^{-4}+\frac{v_{x0}^{2}}{D_{x}B_{z}^{6}}t^{-2}$		${lnD}_{x}B_{z}^{6}t^{2}$		
	y	$\frac{y_{0}^{4}}{D_{y}^{2}}t^{-2}+\frac{y_{0}^{2}}{D_{y}}t^{-1}$	$\frac{y_{0}v_{y0}}{{D_{y}B}_{z}^{3}}t^{-3/2}$	${lnD}_{y}t$	$lnD_{y}^{2}B_{z}^{7}t^{3}$	$\frac{{2D}_{y}^{2}B_{z}^{6}}{3}t^{3}$
	$v_{y}$	$\frac{v_{y0}^{4}}{{D_{y}^{2}B}_{z}^{12}}t^{-4}+\frac{v_{y0}^{2}}{D_{y}B_{z}^{6}}t^{-2}$		${lnD}_{y}B_{z}^{6}t^{2}$		

**Table 2:** Values of the non-Gaussian parameter, the correlation coefficient, the entropy, the combined entropy, and the moments for the motion of a charged particle with two correlated Gaussian forces within the limits of t≪τ, t≫τ and for τ=0, where τ is the correlation time. Here, we assume that a charged particle is initially at $x=x_{0}$, $v_{x}=v_{x0}$ and at ${y=y}_{0}$, $v_{y}=v_{y0}$.

Time	${x,v}_{x}$ ${y,v}_{y}$	${K_{x},K}_{v_{x}}$ ${K_{y},K}_{v_{y}}$	$\rho_{x,v_{x}}$ $\rho_{{y,v}_{y}}$	$S\left(x,t\right),S(v_{x},t)$ $S\left(y,t\right),S(v_{y},t)$	$S({x,v}_{x},t)$ $S({y,v}_{y},t)$	$\mu_{2,2}$, ${\bar{\mu }}_{2,2}$
t≪τ	x	$\frac{x_{0}^{4}}{D_{x}^{2}}t^{-6}+\frac{x_{0}^{2}}{D_{x}}t^{-3}$	$\frac{x_{0}v_{x0}}{{D_{x}B}_{z}^{2}}t^{-3}$	${lnD}_{x}t^{3}$	$lnD_{x}^{2}B_{z}^{4}t^{6}$	$\frac{{2D}_{x}^{2}B_{z}^{4}}{5\tau_{ac}}t^{5}$
	$v_{x}$	$\frac{v_{x0}^{4}}{{D_{x}^{2}B}_{z}^{8}}t^{-6}+\frac{v_{x0}^{2}}{D_{x}B_{z}^{4}}t^{-3}$		${lnD}_{x}B_{z}^{4}t^{3}$		
	y	$\frac{y_{0}^{4}}{D_{y}^{2}}t^{-6}+\frac{y_{0}^{2}}{D_{y}}t^{-3}$	$\frac{y_{0}v_{y0}}{{D_{y}B}_{z}^{2}}t^{-3}$	${lnD}_{y}t^{3}$	$lnD_{y}^{2}B_{z}^{4}t^{6}$	$\frac{{2D}_{y}^{2}B_{z}^{4}}{5\tau_{ac}}t^{5}$
	$v_{y}$	$\frac{v_{y0}^{4}}{{D_{y}^{2}B}_{z}^{8}}t^{-6}+\frac{v_{y0}^{2}}{D_{y}B_{z}^{4}}t^{-3}$		${lnD}_{y}B_{z}^{4}t^{3}$		
t≫τ	x	$\frac{x_{0}^{4}}{D_{x}^{2}B_{z}^{4}}t^{-8}+\frac{x_{0}^{2}}{D_{x}B_{z}^{2}}t^{-4}$	$\frac{x_{0}v_{x0}}{{D_{x}B}_{z}^{3}}t^{-7/2}$	${lnD}_{x}B_{z}^{2}t^{4}$	$lnD_{x}^{2}B_{z}^{6}t^{7}$	$\frac{{2D}_{x}^{2}B_{z}^{4}}{5\tau_{ac}}t^{5}$
	$v_{x}$	$\frac{v_{x0}^{4}}{{D_{x}^{2}B}_{z}^{8}}t^{-6}+\frac{v_{x0}^{2}}{D_{x}B_{z}^{4}}t^{-3}$		${lnD}_{x}B_{z}^{4}t^{3}$		
	y	$\frac{y_{0}^{4}}{D_{y}^{2}B_{z}^{4}}t^{-4}+\frac{y_{0}^{2}}{D_{y}B_{z}^{2}}t^{-4}$	$\frac{y_{0}v_{y0}}{{D_{y}B}_{z}^{3}}t^{-7/2}$	${lnD}_{y}B_{z}^{2}t^{4}$	$lnD_{y}^{2}B_{z}^{6}t^{7}$	$\frac{{2D}_{y}^{2}B_{z}^{4}}{5\tau_{ac}}t^{5}$
	$v_{y}$	$\frac{v_{y0}^{4}}{{D_{y}^{2}B}_{z}^{8}}t^{-6}+\frac{v_{y0}^{2}}{D_{y}B_{z}^{4}}t^{-3}$		${lnD}_{y}B_{z}^{4}t^{3}$		
τ=0	x	$\frac{x_{0}^{4}}{D_{x}^{2}}t^{-6}+\frac{x_{0}^{2}}{D_{x}}t^{-3}$	$\frac{x_{0}v_{x0}}{{D_{x}B}_{z}^{2}}t^{-5/2}$	${lnD}_{x}t^{3}$	$lnD_{x}^{2}B_{z}^{4}t^{5}$	$\frac{D_{x}^{2}B_{z}^{4}}{2\tau_{ac}}t^{4}$
	$v_{x}$	$\frac{v_{x0}^{4}}{{D_{x}^{2}B}_{z}^{8}}t^{-4}+\frac{v_{x0}^{2}}{D_{x}B_{z}^{4}}t^{-2}$		${lnD}_{x}B_{z}^{4}t^{2}$		
	y	$\frac{y_{0}^{4}}{D_{y}^{2}}t^{-6}+\frac{y_{0}^{2}}{D_{y}}t^{-3}$	$\frac{y_{0}v_{y0}}{{D_{y}B}_{z}^{2}}t^{-5/2}$	${lnD}_{y}t^{3}$	$lnD_{y}^{2}B_{z}^{4}t^{5}$	$\frac{D_{y}^{2}B_{z}^{4}}{2\tau_{ac}}t^{4}$
	$v_{y}$	$\frac{v_{y0}^{4}}{{D_{y}^{2}B}_{z}^{8}}t^{-4}+\frac{v_{y0}^{2}}{D_{y}B_{z}^{4}}t^{-2}$		${lnD}_{y}B_{z}^{4}t^{2}$		

**Table 3:** Values of the non-Gaussian parameter, the correlation coefficient, the entropy, the combined entropy, and the moments for the motion of a charged particle with the trap forces and the thermal noises within the limits of t<<τ, t>>τ and for τ=0, where τ is the correlation time. Here, we assume that a charged particle is initially at $x=x_{0}$, $v_{x}=v_{x0}$ and at ${y=y}_{0}$, $v_{y}=v_{y0}$.

Time	${x,v}_{x}$ ${y,v}_{y}$	${K_{x},K}_{v_{x}}$ ${K_{y},K}_{v_{y}}$	$\rho_{x,v_{x}}$ $\rho_{{y,v}_{y}}$	$S\left(x,t\right),S(v_{x},t)$ $S\left(y,t\right),S(v_{y},t)$	$S({x,v}_{x},t)$ $S({y,v}_{y},t)$	$\mu_{2,2}$, ${\bar{\mu }}_{2,2}$
t≪τ	x	$\frac{{\tau_{th}^{4h}x}_{0}^{4}}{D_{x}^{2}}t^{-4h-2}+\frac{{\tau_{th}^{2h}x}_{0}^{2}}{D_{x}}t^{-2h-1}$	$\frac{{\tau_{th}^{2h}x}_{0}v_{x0}}{{D_{x}k}_{1}}t^{-2h-2}$	$ln\frac{D_{x}}{\tau_{th}^{2h}}t^{2h+1}$	$ln\frac{D_{x}^{2}k_{1}^{2}}{\tau_{th}^{4h}}t^{4h+4}$	$\frac{{h^{-1}D}_{x}^{2}k_{1}^{2}}{{(2h+5)\tau }_{th}^{2h-1}}t^{2h+5}$
	$v_{x}$	$\frac{{\tau_{th}^{4h}v}_{x0}^{4}}{D_{x}^{2}k_{1}^{4}}t^{-4h-6}+\frac{\tau_{th}^{2h}v_{x0}^{2}}{D_{x}k_{1}^{2}}t^{-2h-3}$		$ln\frac{D_{x}k_{1}^{2}}{\tau_{th}^{2h}}t^{2h+3}$		
	y	$\frac{{\tau_{th}^{4h}y}_{0}^{4}}{D_{y}^{2}}t^{-4h-2}+\frac{{\tau_{th}^{2h}y}_{0}^{2}}{D_{y}}t^{-2h-1}$	$\frac{{\tau_{th}^{2h}y}_{0}v_{y0}}{{D_{y}k}_{2}B_{z}}t^{-2h-2}$	$ln\frac{D_{y}}{\tau_{th}^{2h}}t^{2h+1}$	$ln\frac{D_{y}^{2}k_{2}^{2}}{\tau_{th}^{4h}}t^{4h+4}$	$\frac{h^{-1}D_{y}^{2}k_{2}^{2}}{{(2h+5)\tau }_{th}^{2h-1}}t^{2h+5}$
	$v_{y}$	$\frac{{\tau_{th}^{4h}v}_{y0}^{4}}{D_{y}^{2}k_{2}^{4}}t^{-4h-6}+\frac{\tau_{th}^{2h}v_{y0}^{2}}{D_{y}k_{2}^{2}}t^{-2h-3}$		$ln\frac{D_{y}k_{2}^{2}}{\tau_{th}^{2h}}t^{2h+3}$		
t≫τ	x	$\frac{{\tau_{th}^{4h-2}x}_{0}^{4}}{D_{x}^{2}}t^{-4h-2}+\frac{{\tau_{th}^{2h-1}x}_{0}^{2}}{D_{x}}t^{-2h-1}$	$\frac{{\tau_{th}^{2h-1/2}x}_{0}v_{x0}}{{D_{x}k}_{1}B_{z}}t^{-2h-2}$	$ln\frac{D_{x}}{\tau_{th}^{2h-1}}t^{2h+1}$	$ln\frac{D_{x}^{2}k_{1}^{2}}{\tau_{th}^{4h-1}}t^{4h+4}$	$\frac{{h^{-1}D}_{x}^{2}k_{1}^{2}}{{(2h+5)\tau }_{th}^{2h-1}}t^{2h+5}$
	$v_{x}$	$\frac{{\tau_{th}^{4h}v}_{x0}^{4}}{D_{x}^{2}k_{1}^{4}}t^{-4h-6}+\frac{\tau_{th}^{2h}v_{x0}^{2}}{D_{x}k_{1}^{2}}t^{-2h-3}$		$ln\frac{D_{x}k_{1}^{2}}{\tau_{th}^{2h}}t^{2h+3}$		
	y	$\frac{{\tau_{th}^{4h-2}y}_{0}^{4}}{D_{y}^{2}}t^{-4h-2}+\frac{{\tau_{th}^{2h-1}y}_{0}^{2}}{D_{y}}t^{-2h-1}$	$\frac{{\tau_{th}^{2h-1/2}y}_{0}v_{y0}}{{D_{y}k}_{2}B_{z}}t^{-2h-2}$	$ln\frac{D_{x}}{\tau_{th}^{2h-1}}t^{2h+2}$	$ln\frac{D_{y}^{2}k_{2}^{2}}{\tau_{th}^{4h-1}}t^{4h+4}$	$\frac{h^{-1}D_{y}^{2}k_{2}^{2}}{{(2h+5)\tau }_{th}^{2h-1}}t^{2h+5}$
	$v_{y}$	$\frac{{\tau_{th}^{4h}v}_{y0}^{4}}{D_{y}^{2}k_{2}^{4}}t^{-4h-6}+\frac{\tau_{th}^{2h}v_{y0}^{2}}{D_{y}k_{2}^{2}}t^{-2h-3}$		$ln\frac{D_{y}k_{2}^{2}}{\tau_{th}^{2h}}t^{2h+3}$		
τ=0	x	$\frac{{\tau_{th}^{4h}x}_{0}^{4}}{D_{x}^{2}}t^{-4h-2}+\frac{{\tau_{th}^{2h}x}_{0}^{2}}{D_{x}}t^{-2h-1}$	$\frac{{\tau_{th}^{2h}x}_{0}v_{x0}}{{D_{x}k}_{1}}t^{-2h-2}$	$ln\frac{D_{x}}{\tau_{th}^{2h}}t^{2h+1}$	$ln\frac{D_{x}^{2}k_{1}^{2}}{\tau_{th}^{4h}}t^{4h+4}$	$\frac{{h^{-1}D}_{x}^{2}k_{1}^{2}}{{(2h+5)\tau }_{th}^{2h-1}}t^{2h+5}$
	$v_{x}$	$\frac{{\tau_{th}^{2h}v}_{x0}^{4}}{D_{x}^{2}k_{1}^{4}}t^{-4h-6}+\frac{\tau_{th}^{2h}v_{x0}^{2}}{D_{x}k_{1}^{2}}t^{-2h-3}$		$ln\frac{D_{x}k_{1}^{2}}{\tau_{th}^{2h}}t^{2h+3}$		
	y	$\frac{{\tau_{th}^{4h}y}_{0}^{4}}{D_{y}^{2}}t^{-4h-2}+\frac{{\tau_{th}^{2h}y}_{0}^{2}}{D_{y}}t^{-2h-1}$	$\frac{{\tau_{th}^{2h}y}_{0}v_{y0}}{{D_{y}k}_{2}}t^{-2h-2}$	$ln\frac{D_{x}B_{z}^{2}}{\tau_{th}^{2h}}t^{2h+2}$	$ln\frac{D_{y}^{2}k_{2}^{2}}{\tau_{th}^{4h}}t^{4h+4}$	$\frac{h^{-1}D_{y}^{2}k_{2}^{2}}{{(2h+5)\tau }_{th}^{2h-1}}t^{2h+5}$
	$v_{y}$	$\frac{{\tau_{th}^{2h}v}_{y0}^{4}}{D_{y}^{2}k_{2}^{4}}t^{-4h-6}+\frac{\tau_{th}^{2h}v_{y0}^{2}}{D_{y}k_{2}^{2}}t^{-2h-3}$		$ln\frac{D_{y}k_{2}^{2}}{\tau_{th}^{2h}}t^{2h+3}$		

**Table 4:** Values of the non-Gaussian parameter, the correlation coefficient, the entropy, the combined entropy, and the moments for the motion of a charged particle with the trap forces and the active noises within the limits of t<<τ, t>>τ, and for τ=0, where τ is the correlation time. Here, we assume that a charged particle is initially at $x=x_{0}$, $v_{x}=v_{x0}$ and at ${y=y}_{0}$, $v_{y}=v_{y0}$.

Time	${x,v}_{x}$ ${y,v}_{y}$	${K_{x},K}_{v_{x}}$ ${K_{y},K}_{v_{y}}$	$\rho_{x,v_{x}}$ $\rho_{{y,v}_{y}}$	$S\left(x,t\right),S(v_{x},t)$ $S\left(y,t\right),S(v_{y},t)$	$S({x,v}_{x},t)$ $S({y,v}_{y},t)$	$\mu_{2,2}$, ${\bar{\mu }}_{2,2}$
t≪τ	x	$\frac{{k_{1}^{2}x}_{0}^{4}}{D_{x}^{2}}t^{-6}+\frac{k_{1}x_{0}^{2}}{D_{x}}t^{-3}$	$\frac{{k_{1}x}_{0}v_{x0}}{D_{x}B_{z}^{2}}t^{-3}$	$ln\frac{D_{x}}{k_{1}}t^{3}$	$ln\frac{D_{x}^{2}B_{z}^{4}}{k_{1}^{2}}t^{6}$	$\frac{{2D}_{x}^{2}B_{z}^{4}}{5k_{1}\tau_{ac}}t^{5}$
	$v_{x}$	$\frac{{k_{1}^{2}v}_{x0}^{4}}{D_{x}^{2}B_{z}^{8}}t^{-6}+\frac{k_{1}v_{x0}^{2}}{D_{x}B_{z}^{4}}t^{-3}$		$ln\frac{D_{x}B_{z}^{4}}{k_{1}}t^{3}$		
	y	$\frac{{k_{2}^{2}y}_{0}^{4}}{D_{y}^{2}}t^{-6}+\frac{k_{2}y_{0}^{2}}{D_{y}}t^{-3}$	$\frac{{k_{2}y}_{0}v_{y0}}{D_{y}B_{z}^{2}}t^{-3}$	$ln\frac{D_{y}}{k_{2}}t^{3}$	$ln\frac{D_{y}^{2}B_{z}^{4}}{k_{2}^{2}}t^{6}$	$\frac{{2D}_{y}^{2}B_{z}^{4}}{5k_{2}\tau_{ac}}t^{5}$
	$v_{y}$	$\frac{k_{2}^{2}v_{y0}^{4}}{D_{y}^{2}B_{z}^{8}}t^{-6}+\frac{k_{2}v_{y0}^{2}}{D_{y}B_{z}^{4}}t^{-3}$		$ln\frac{D_{y}B_{z}^{4}}{k_{2}}t^{3}$		
t≫τ	x	$\frac{{k_{1}^{2}x}_{0}^{4}}{D_{x}^{2}}t^{-6}+\frac{k_{1}x_{0}^{2}}{D_{x}}t^{-3}$	$\frac{{k_{1}x}_{0}v_{x0}}{D_{x}B_{z}^{2}}t^{-3}$	$ln\frac{D_{x}}{k_{1}}t^{3}$	$ln\frac{D_{x}^{2}B_{z}^{4}}{k_{1}^{2}}t^{6}$	$\frac{{2D}_{x}^{2}B_{z}^{4}}{5k_{1}\tau_{ac}}t^{5}$
	$v_{x}$	$\frac{{k_{1}^{2}v}_{x0}^{4}}{D_{x}^{2}B_{z}^{8}}t^{-6}+\frac{k_{1}v_{x0}^{2}}{D_{x}B_{z}^{4}}t^{-3}$		$ln\frac{D_{x}B_{z}^{4}}{k_{1}}t^{3}$		
	y	$\frac{{k_{2}^{2}y}_{0}^{4}}{D_{y}^{2}}t^{-6}+\frac{k_{2}y_{0}^{2}}{D_{y}}t^{-3}$	$\frac{{k_{2}y}_{0}v_{y0}}{D_{y}B_{z}^{2}}t^{-3}$	$ln\frac{D_{y}}{k_{2}}t^{3}$	$ln\frac{D_{y}^{2}B_{z}^{4}}{k_{2}^{2}}t^{6}$	$\frac{{2D}_{y}^{2}B_{z}^{4}}{5k_{2}\tau_{ac}}t^{5}$
	$v_{y}$	$\frac{k_{2}^{2}v_{y0}^{4}}{D_{y}^{2}B_{z}^{8}}t^{-6}+\frac{k_{2}v_{y0}^{2}}{D_{y}B_{z}^{4}}t^{-3}$		$ln\frac{D_{y}B_{z}^{4}}{k_{2}}t^{3}$		
τ=0	x	$\frac{x_{0}^{4}}{D_{x}^{2}}t^{-6}+\frac{x_{0}^{2}}{D_{x}}t^{-3}$	$\frac{x_{0}v_{x0}}{D_{x}}t^{-2}$	$lnD_{x}t^{3}$	$lnD_{x}^{2}t^{4}$	$\frac{{2D}_{x}^{2}}{3\tau_{ac}}t^{3}$
	$v_{x}$	$\frac{v_{x0}^{4}}{D_{x}^{2}}t^{-2}+\frac{v_{x0}^{2}}{D_{x}}t^{-1}$		$lnD_{x}t$		
	y	$\frac{y_{0}^{4}}{D_{y}^{2}}t^{-6}+\frac{y_{0}^{2}}{D_{y}}t^{-3}$	$\frac{y_{0}v_{y0}}{D_{y}B_{z}^{2}}t^{-2}$	$lnD_{y}t^{3}$	$lnD_{y}^{2}t^{4}$	$\frac{{2D}_{y}^{2}}{3\tau_{ac}}t^{3}$
	$v_{y}$	$\frac{v_{y0}^{4}}{D_{y}^{2}}t^{-2}+\frac{v_{y0}^{2}}{D_{y}}t^{-1}$		$lnD_{y}t$		

## Data Availability

Data will be made available upon request.

## Appendix A. Derivation of the Time Derivative of the Joint Probability Density

The equations of motion for a charged particle in a magnetic field $\vec{B}=B_{z}\hat{z}$ are given by

(A1) $$\frac{\partial }{\partial t}x=v_{x}{+\eta }_{x}\left(t\right),\;\;m\frac{\partial }{\partial t}v_{x}=-qv_{y}B_{z},$$

(A2) $$\frac{\partial }{\partial t}y=v_{y}{+\eta }_{y}\left(t\right),\;m\frac{\partial }{\partial t}v_{y}=+qv_{x}B_{z}.$$

Here, the random forces $\eta_{x}(t)$ and $\eta_{y}(t)$ depend on the time difference and satisfy $<\eta_{i}\left(t\right)\eta_{i}\left(t^{\prime }\right)>=\frac{D_{i}}{\tau }\delta (\frac{\left|t-t\prime \right|}{\tau })$ for i=x,y. The joint probability density for the displacement (x,y) and the velocity ${(v}_{x},v_{y})$ is defined by $f(x,v_{x},y,v_{y},t)=<\delta (x-x(t))\delta (v_{x}-v_{x}(t)\delta (y-y(t))\delta (v_{y}-v_{y}(t))>$. Taking time derivative of the joint probability density yields

(A3) $$\frac{\partial }{\partial t}f=-\frac{\partial }{\partial x}<\frac{\partial x}{\partial t}\delta_{x}\delta_{v_{x}}\delta_{y}\delta_{v_{y}}>-\frac{\partial }{\partial v_{x}}<\frac{\partial v_{x}}{\partial t}\delta_{x}\delta_{v_{x}}\delta_{y}\delta_{v_{y}}>-\frac{\partial }{\partial y}<\frac{\partial y}{\partial t}\delta_{x}\delta_{v_{x}}\delta_{y}\delta_{v_{y}}>-\frac{\partial }{\partial v_{y}}<\delta_{x}\delta_{v_{x}}\delta_{y}\delta_{v_{y}}>,$$

where $f\left(x,v_{x},y,v_{y},t\right)=f$, $\delta_{x}=\delta (x-x\left(t\right)),\delta_{v_{x}}=\delta (v_{x}-v_{x}\left(t\right)),\delta_{y}=\delta (y-y\left(t\right)),$ and $\delta_{v_{y}}=\delta (v_{y}-v_{y}\left(t\right)$. Substituting Equations (3) and (4) into Equation (5) and manipulating the method of Ref. [13], we obtain

(A4) $$\frac{\partial }{\partial t}f+v_{x}\frac{\partial }{\partial x}f+v_{y}\frac{\partial }{\partial y}f+\frac{q}{m}v_{y}B_{z}\frac{\partial }{\partial v_{x}}f-\frac{q}{m}v_{x}B_{z}\frac{\partial }{\partial v_{y}}f=D_{x}{\nabla_{x}}^{2}f+D_{y}{\nabla_{y}}^{2}f.$$

Equation (A4) thus represents the time evolution equation for the joint probability density, corresponding to Equation (2) in the main text.

## Appendix B. Derivation of the Time Evolution Equation for the Joint Probability Density

The joint probability densities $f(x,v_{x},t)$ and $f(y,v_{y},t)$ for the displacement (x,y) and the velocity $\left(v_{x},v_{y}\right)$ are defined by $f(x,v_{x},t)=<\delta (x-x(t))\delta (v_{x}-v_{x}(t))>$ and $f(y,v_{y},t)=<\delta (y-y(t))\delta (v_{y}-v_{y}(t))>$. Taking time derivatives of the joint probability density yields

(A5) $$\frac{\partial }{\partial t}f(x,v_{x},t)=-\frac{\partial }{\partial x}<\frac{\partial x}{\partial t}\delta (x-x(t))\delta (v_{x}-v_{x}(t))>-\frac{\partial }{\partial v_{x}}<\frac{\partial v_{x}}{\partial t}\delta (x-x(t))\delta (v_{x}-v_{x}(t))>,$$

(A6) $$\frac{\partial }{\partial t}f\left(y,v_{y},t\right)=-\frac{\partial }{\partial y}<\frac{\partial y}{\partial t}\delta \left(y-y\left(t\right)\right)\delta \left(v_{y}-v_{y}\left(t\right)\right)>-\frac{\partial }{\partial v_{y}}<\frac{\partial v_{y}}{\partial t}\delta \left(y-y\left(t\right)\right)\delta \left(v_{y}-v_{y}\left(t\right)\right)>.$$

Applying the Laplace transforms of Equations (3) and (4), Equation (3) becomes

(A7) $$sv_{x}\left(s\right)-v_{x0}={-B}_{z}v_{y}(s){-r_{1}v}_{x}(s),$$

and Equation (4) yields

(A8) $$sv_{y}\left(s\right)-v_{y0}={+B}_{z}v_{x}\left(s\right){-r_{2}v}_{y}(s).$$

Here, the initial condition is $x\left(0\right)=x_{0},\;v_{x}\left(0\right)=v_{x0}$ and $x\left(0\right)=x_{0},\;v_{x}\left(0\right)=v_{x0}$ and L denotes the Laplace transform operator. Solving Equations (A7) and (A8), we obtain

(A9) $$v_{x}\left(s\right)=\frac{v_{x0}}{s+r_{1}}-\frac{B_{z}}{s+r_{1}}v_{y}(s),\;$$

(A10) $$v_{y}\left(s\right)=\frac{v_{y0}}{s+r_{2}}+\frac{B_{z}}{s+r_{2}}v_{x}(s).$$

Taking the inverse Laplace transform, we find

(A11) $$v_{x}\left(t\right)=v_{x0}exp(-r_{1}t)-B_{z}\int dt^{\prime }exp[-r_{1}(t-t^{\prime })]v_{y}(t\prime ),\;$$

(A12) $$v_{y}\left(t\right)=v_{y0}\exp\left(-r_{2}t\right)+B_{z}\int dt^{\prime }exp[-r_{2}(t-t^{\prime })]v_{x}(t\prime ).$$

Consequently, substituting Equations (A11) and (A12) into Equations (28) and (29) and rearranging Equations (28) and (29), we obtain the time evolution equations for $f\left(x,v_{x},t\right)$ and $f\left(y,v_{y},t\right)$, given by Equations (30) and (31).
